# Magnesium sulfate pharmacology for maternal and critical-care indications: mechanisms, pharmacokinetics, and the therapeutic window

**DOI:** 10.3389/fphar.2026.1749828

**Published:** 2026-02-20

**Authors:** Mingya Xia, Qiang Ni, Sha Zhu

**Affiliations:** 1 West China Second University Hospital, Sichuan University, Chengdu, China; 2 Key Laboratory of Birth Defects and Related Diseases of Women and Children (Sichuan University), Ministry of Education, Chengdu, China

**Keywords:** antenatal neuroprotection, hypertensive disorders of pregnancy, implementation science, magnesium sulfate, maternal safety bundles, obstetric critical care, pharmacokinetics and dosing, preeclampsia and eclampsia

## Abstract

Hypertensive disorders of pregnancy and related critical illnesses remain leading global causes of maternal and perinatal morbidity, and magnesium sulfate is now a cornerstone therapy across obstetric, perinatal, and critical-care settings. However, its use in practice remains heterogeneous. This narrative, practice-oriented review synthesizes mechanistic, pharmacologic, clinical, and implementation evidence on magnesium sulfate use in pregnancy and critical illness. We draw on mechanistic and pharmacokinetic studies, randomized and observational clinical research, guidelines, and quality-improvement reports, emphasizing literature from 2020 to 2025 and selectively incorporating earlier landmark trials and classic pharmacology papers. We describe the multi-mechanistic actions that underpin anticonvulsant and neuroprotective effects, summarize pharmacokinetic variability and exposure targets, and appraise efficacy and safety across key indications, including prevention and treatment of eclampsia and severe preeclampsia, antenatal neuroprotection before very preterm birth, treatment of acute severe hypertension, and perioperative or critical-care adjunct use. We highlight how renal function, body size, and co-administered sedative or neuromuscular blocking agents shape dosing, toxicity risk, and monitoring strategies, and we contrast clinical examination–led and laboratory-led approaches to safety surveillance. At the systems level, we synthesize evidence on digital order sets, early-warning tools, remote postpartum blood pressure monitoring, and equity-stratified quality bundles designed to close gaps in timely treatment and safe monitoring, particularly in low-resource settings. Remaining gaps include precision dosing in special populations, long-term outcomes after antenatal exposure, and formal economic evaluations of care bundles that incorporate magnesium sulfate. Collectively, this review reframes magnesium sulfate as part of an integrated maternal and critical-care safety bundle and proposes a research agenda that links pharmacology, clinical trials, and implementation science to safer and more equitable use.

## 1 Introduction

Hypertensive disorders of pregnancy (HDP)—including gestational hypertension, preeclampsia, eclampsia, and related entities such as HELLP (hemolysis, elevated liver enzymes, low platelets) syndrome—remain a leading cause of maternal and perinatal morbidity and mortality worldwide (World Health Organization, 2025). Recent global estimates indicate that preeclampsia affects 2%–8% of pregnancies and is associated with approximately 46,000 maternal deaths and 500,000 fetal or newborn deaths each year, with disproportionate contributions from Asia, Africa, and Latin America (World Health Organization, 2025). Using Global Burden of Disease 2021 data, a 1990–2021 analysis reported persistently high disability-adjusted life-year losses from maternal hypertensive disorders (age-standardized disability-adjusted life-year rate ∼31 per 100,000 in 2021) and wide regional disparities, with the highest burden in low–Sociodemographic Index settings (Hu et al., 2025). Beyond mortality, HDP increases the risks of placental abruption, fetal growth restriction, preterm birth, stroke, and future cardiometabolic disease for the mother (World Health Organization, 2025; Jiang et al., 2022), underscoring the continuing need for optimized prevention and acute seizure prophylaxis and treatment. Importantly, access to magnesium sulfate (MgSO_4_) and to reliable monitoring remains uneven across health systems, with implementation barriers documented in many low- and middle-income countries (LMICs) (Eddy et al., 2022).

The modern era of MgSO_4_ therapy was catalyzed by landmark randomized evidence in the late 1990s and early 2000s—including the Magnesium Sulphate for Prevention of Eclampsia trial—which demonstrated that MgSO_4_ more than halves the risk of eclampsia *versus* placebo and is superior to alternatives such as diazepam or phenytoin (historical landmark) (Altman et al., 2002). Contemporary guidelines now endorse MgSO_4_ as first-line therapy for prevention and treatment of eclamptic seizures and for severe features of preeclampsia, with routine bedside monitoring of deep tendon reflexes, respiratory rate, and urine output, and dose adjustment in renal impairment (World Health Organization, 2025; Gestational Hypertension and Preeclampsia: ACOG Practice Bulletin, 2020).

Indications for MgSO_4_ have expanded beyond eclampsia. For fetal neuroprotection, guidelines recommend antenatal MgSO_4_ when preterm birth is imminent, with the greatest certainty of benefit at earlier gestational ages (GA); National Institute for Health and Care Excellence advises “offer” between 24 + 0 and 29 + 6 weeks and “consider” between 30 + 0 and 33 + 6 weeks when birth within 24 h is likely (National Institute for Health and Car e Excellence NICE, 2015). Implementation programs such as Prevention of Cerebral Palsy in PreTerm Labour in England have increased MgSO_4_ uptake for threatened preterm birth and are associated with favorable cost-effectiveness and sustained practice change at scale (Edwards et al., 2025). In critical-care (ICU) and emergency contexts, intravenous (IV) MgSO_4_ is recommended for severe acute asthma with inadequate response to initial bronchodilators and corticosteroids, reflecting bronchodilation *via* calcium-channel effects and smooth-muscle relaxation (Global Initiative for Asthma GINA, 2025). Perioperatively, MgSO_4_’s noncompetitive N-methyl-D-aspartate (NMDA) receptor antagonism and calcium-channel effects can attenuate central sensitization, lower anesthetic requirements, and reduce postoperative opioid consumption; in cesarean delivery, meta-analyses and randomized trials demonstrate opioid-sparing effects and early reductions in pain scores, although effect sizes vary and obstetric-specific trials remain limited (Ma et al., 2022). Selected obstetric and perinatal procedures also use MgSO_4_ to facilitate uterine relaxation, but prolonged or repeated courses for tocolysis are discouraged owing to neonatal skeletal adverse effects; regulators advise against more than 5–7 days of MgSO_4_ for preterm labor (U .S. Food and Drug Administration FDA, 2013). These evolving indications highlight both therapeutic breadth and areas of controversy (e.g., optimal dosing across indications, uterine relaxation *versus* atony risk, and context-specific benefit at later GA for neuroprotection).

A new comprehensive review is timely for several reasons. First, dosing and safety insights have matured: pharmacologic syntheses emphasize variability in serum magnesium levels with standard regimens, the narrow therapeutic window for anticonvulsant effects, and the need for individualized dosing in renal impairment (Gestational Hypertension and Preeclampsia: ACOG Practice Bulletin, 2020; Deng et al., 2024). Second, mechanistic understanding—spanning NMDA antagonism, cerebral vasodilation, endothelial stabilization, and anticonvulsant properties—has been integrated with contemporary clinico-radiologic descriptions of eclampsia (e.g., posterior reversible encephalopathy syndrome, cerebrovascular autoregulation failure) (Fishel Bartal and Sibai, 2022). Third, monitoring technologies and pragmatic protocols have simplified bedside assessment and toxicity prevention in diverse settings, yet real-world implementation remains uneven, necessitating an equity lens that addresses supply chains, nurse-led protocols, and escalation pathways in LMIC facilities (Eddy et al., 2022; Magee et al., 2022a). Fourth, emerging randomized controlled trials (RCTs) and meta-analyses continue to refine perioperative and critical-care uses, quantify opioid-sparing effects, and clarify where MgSO_4_ adds value alongside modern multimodal regimens (Ma et al., 2022; Global Initiative for Asthma GINA, 2024). Finally, implementation-science evaluations of national neuroprotection programs provide practice-based evidence on uptake, fidelity, and cost-effectiveness that can inform scale-up beyond high-income countries (Edwards et al., 2025).

In this narrative review, we first summarize pharmacology and putative mechanisms relevant to obstetrics, anesthesia/ICU, and perinatal neurology (including NMDA receptor–related excitability modulation, calcium-channel and vasodilatory effects) to ground dose–response and toxicity considerations. We then appraise clinical efficacy and safety across core indications: prevention and treatment of eclampsia and severe preeclampsia; fetal neuroprotection by GA band; ICU and emergency indications (e.g., severe asthma); and perioperative and obstetric-anesthesia applications (including opioid-sparing and uterine relaxation), highlighting areas of consensus and controversy. Special populations (renal impairment, extremes of body size, limited-resource settings) are considered with pragmatic monitoring guidance. Next, we examine implementation and equity—covering supply, training, and quality-improvement programs that close the “know–do” gap in LMICs—and conclude with future directions: model-informed dosing, point-of-care monitoring, and targeted trials to define benefit–risk by indication and GA.

## 2 Methods

This article is a narrative, practice-oriented review that synthesizes evidence on the mechanisms, pharmacology, clinical indications, toxicity, and systems-level implementation of MgSO_4_ in obstetric and critical-care practice. Our aim was not to conduct a formal meta-analysis, but to integrate key randomized trials, pharmacokinetic–pharmacodynamic (PK–PD) studies, observational data, guidelines, and quality-improvement (QI) initiatives into a coherent framework for contemporary use.

We focused primarily on literature published between January 2020 and October 2025, while selectively including earlier landmark trials and classic pharmacology papers when they were essential for current practice or mechanistic understanding. Electronic searches were performed in PubMed/MEDLINE, Embase, Web of Science, and the Cochrane Library using combinations of keywords and Medical Subject Headings terms related to magnesium sulfate and its main clinical contexts (e.g., “magnesium sulfate” AND “preeclampsia,” “eclampsia,” “hypertensive disorders of pregnancy,” “neuroprotection,” “preterm birth,” “anesthesia,” “analgesia,” “intensive care,” “toxicity,” “quality improvement”). To capture guidance and implementation models, we also reviewed documents and toolkits from major professional societies and organizations (obstetrics, anesthesia, nephrology, neurology), national and international guideline groups, perinatal quality collaboratives, and global quality-of-care networks (QCNs), and hand-searched reference lists of key articles and reviews.

Eligibility criteria were intentionally broad but prespecified. We included: (i) mechanistic and PK–PD studies of MgSO_4_; (ii) randomized and nonrandomized clinical studies in pregnant or postpartum populations (e.g., preeclampsia/eclampsia, hypertensive emergencies, threatened preterm birth); (iii) perioperative and ICU studies in obstetric patients and, when directly informative, in nonobstetric patients; and (iv) guidelines, consensus statements, QI reports, and implementation case studies addressing MgSO_4_ dosing, monitoring, safety, or bundle design. Case reports and small case series were used selectively to illustrate rare toxicity patterns or unusual indications. Non-English publications were considered when they provided unique data or influential recommendations.

Titles and abstracts were screened for relevance to the review aims, and full texts were retrieved for potentially eligible articles. From each source, we extracted data on setting and population, MgSO_4_ regimen (route, loading and maintenance doses, duration), comparators, outcomes (e.g., seizure recurrence, maternal and neonatal outcomes, PK–PD parameters, toxicity), and key implementation or safety findings. Given the heterogeneity in study designs, populations, and outcome definitions, we did not attempt a quantitative meta-analysis. Instead, findings were organized thematically: molecular and cellular mechanisms (Section 3); PK–PD and the therapeutic window (Section 4); clinical indications and regimens (Section 5); toxicity and monitoring (Section 6); systems-level implementation and equity (Section 7); major controversies (Section 8); and future research and practice implications (Sections 9–11).

## 3 Fundamental concepts and mechanisms of action

### 3.1 Molecular pharmacology

MgSO_4_ acts primarily through the magnesium ion (Mg^2+^), a physiological, voltage-dependent open-channel blocker of NMDA receptors that reduces calcium influx during excitatory synaptic transmission and raises the seizure threshold by dampening glutamatergic drive (Magee et al., 2022b; Wilcox et al., 2022). At resting membrane potentials, Mg^2+^ occupies the NMDA receptor pore and is expelled during depolarization; therapeutically increasing extracellular Mg^2+^ strengthens this block and helps stabilize cortical networks (Magee et al., 2022b; Wilcox et al., 2022). Beyond NMDA receptors, Mg^2+^ modulates voltage-gated calcium channels—particularly L-type (CaV1.x) channels on vascular smooth muscle and presynaptic N-/P/Q-type (CaV2.x) channels—thereby reducing presynaptic Ca^2+^ entry and neurotransmitter release while promoting vasorelaxation (Catterall, 2023; Ch et al., 2022).

At the neuromuscular junction, Mg^2+^ inhibits presynaptic acetylcholine release and slightly decreases postsynaptic sensitivity, which clinically potentiates nondepolarizing neuromuscular blocking agents. Meticulous quantitative neuromuscular monitoring and appropriate reversal are therefore recommended when perioperative or ICU MgSO_4_ exposure coexists with nondepolarizing neuromuscular blocking agents (Thilen et al., 2023; Dahake et al., 2024).

Clinically, the ionized fraction of magnesium (iMg^2+^) is the active moiety, whereas total serum magnesium correlates imperfectly with iMg^2+^ in critical illness. Renal handling—filtration and reabsorption primarily in the thick ascending limb—and acid–base status influence iMg^2+^ availability (Adomako and Yu, 2024). Limited, historical human data show only modest increases in cerebrospinal fluid magnesium after IV MgSO_4_; because cerebrospinal fluid magnesium is an imperfect surrogate for synaptic/interstitial exposure, these observations are best interpreted as consistent with central nervous system effects arising from enhanced synaptic excitability control (e.g., NMDA/Ca^2+^-linked mechanisms) together with cerebrovascular/endothelial actions, rather than from large bulk blood–brain barrier transfer (Thurnau et al., 1987).

Beyond these proximal electrophysiologic effects, MgSO_4_ has been shown to modulate intracellular inflammatory signaling and transcriptional outputs relevant to endothelial activation. In human umbilical vein endothelial cells, MgSO_4_ reduced nuclear factor kappa B (NF-κB) nuclear translocation and preserved inhibitor of κB alpha, with downstream suppression of interleukin-8 release and intercellular adhesion molecule 1 expression after lipopolysaccharide stimulation (Rochelson et al., 2007). In innate immune and neuroinflammation models, clinically relevant MgSO_4_ exposure reduced pro-inflammatory cytokine production (including tumor necrosis factor alpha and interleukin-6) and was associated with increased basal inhibitor of κB alpha and reduced NF-κB activation; in lipopolysaccharide-activated primary microglia, MgSO_4_ inhibited NF-κB translocation and decreased interleukin-1 beta (IL-1β) and tumor necrosis factor alpha release (with concurrent reductions in nitric oxide/prostaglandin E2) (Sugimoto et al., 2012; Gao et al., 2013). In pregnancy-specific models, MgSO_4_ suppressed placental NF-κB–linked inflammation in a lipopolysaccharide-induced preeclampsia-like rat model (including reduced placental IL-1β/interleukin-12 with improved placental function and angiogenesis signals) and attenuated excessive placental cytokine secretion in *ex vivo* perfusion studies of preeclamptic placentas (e.g., reduced maternal-side IL-1β secretion and altered interleukin-1 receptor antagonist dynamics) (Wu et al., 2023; Amash et al., 2012).

At the organelle level, MgSO_4_ has been linked to mitochondrial protection and apoptosis-pathway modulation: in hypoxia-stress neuronal models, MgSO_4_ preserved mitochondrial membrane potential, reduced cytochrome-c release, increased anti-apoptotic B-cell lymphoma 2 family proteins, and engaged mitogen-activated protein kinase signaling (extracellular signal–regulated kinase 1/2 activation with attenuation of p38 mitogen-activated protein kinase/c-Jun N-terminal kinase activity), consistent with improved cellular resilience (Huang et al., 2015; Huang et al., 2010). Consistent with bioenergetic relevance, MgSO_4_ reduced brain-mitochondria oxidative damage after experimental hypoxia and improved mitochondrial respiratory function indices in brain-injury models (Mohammadi et al., 2020; Xu et al., 2002). Given oxidative stress as a key driver in placental pathology, we additionally note nuclear factor erythroid 2–related factor 2-centered antioxidant transcription as a plausible integrating axis; in a pregnancy rat model, MgSO_4_ increased placental nuclear factor erythroid 2–related factor 2 (and antioxidant proteins such as peroxiredoxin 6) while reducing inflammatory cytokines (e.g., IL-1β, tumor necrosis factor alpha, interferon-gamma), supporting biologic plausibility that MgSO_4_ can couple anti-inflammatory and antioxidant programs, although direct confirmation in preeclampsia tissues remains limited (Han et al., 2018). Finally, angiogenic signaling can also be MgSO_4_-responsive: placental perfusion data suggest MgSO_4_ can alter vascular endothelial growth factor expression in a compartment- and phenotype-dependent manner, and placental ischemia models demonstrate reductions in cerebrospinal fluid vascular endothelial growth factor alongside cytokine/chemokine attenuation with MgSO_4_ (Weintraub et al., 2013; Zhang and Warrington, 2016). To date, pregnancy-tissue–specific data directly interrogating MgSO_4_ effects on canonical transforming growth factor beta/SMAD signaling are sparse; targeted mechanistic studies are therefore warranted. For an overview of preeclampsia pathophysiology and adverse outcomes during pregnancy and postpartum, see Bisson et al. (2023).

### 3.2 System-level actions

#### 3.2.1 Anticonvulsant effects in eclampsia

In eclampsia, MgSO_4_ reduces seizure recurrence and is guideline-endorsed as the anticonvulsant of choice, plausibly through multisite network stabilization (NMDA antagonism), improved cerebrovascular autoregulation, and endothelial effects (World Health Organization, 2025; Magee et al., 2022b; Rochelson et al., 2007).

#### 3.2.2 Vasodilatory/endothelial effects

Mg^2+^ promotes systemic and cerebral vasodilation (in part *via* L-type channel modulation and nitric oxide–linked endothelial pathways) and may counteract the vasoconstriction and endothelial activation central to preeclampsia and HELLP syndrome pathophysiology (National Institute for Health and Car e Excellence NICE, 2015; Magee et al., 2022b). These vascular actions also help explain blood pressure “smoothing” and fewer cerebral hyperperfusion spikes during severe HDP (National Institute for Health and Car e Excellence NICE, 2015; Magee et al., 2022b).

#### 3.2.3 Neuroprotection of the preterm fetus

Antenatal MgSO_4_ given before imminent early preterm birth reduces cerebral palsy (CP) and the composite of death or CP; putative mechanisms include NMDA-related anti-excitotoxic effects, modulation of neuroinflammation/oxidative stress, and cerebrovascular stabilization, but these mechanistic explanations remain hypothesized and are largely inferred from preclinical and translational evidence. Contemporary systematic reviews and guidelines support its use, particularly at lower GA (World Health Organization, 2025; Rochelson et al., 2007; Shepherd et al., 2024).

#### 3.2.4 Uterine effects

Mg^2+^ relaxes myometrium *in vitro via* Ca^2+^ antagonism, but clinical tocolytic effects are inconsistent. Modern guidance prioritizes MgSO_4_ for fetal neuroprotection rather than as a routine tocolytic, reflecting uncertain benefit–risk for delaying birth (National Institute for Health and Car e Excellence NICE, 2015; Shepherd et al., 2024).

### 3.3 Pregnancy-specific physiology affecting Mg^2+^ homeostasis

Pregnancy alters Mg^2+^ pharmacokinetics through hemodilution, increased glomerular filtration rate (GFR), and changes in gastrointestinal absorption and volume of distribution across maternal–fetal compartments. Placental transfer occurs, with fetal exposure directly relevant to neuroprotection (Magee et al., 2022b). Population-based and clinical data emphasize that reduced renal clearance elevates toxicity risk, whereas augmented clearance in late gestation may lower total serum magnesium and iMg^2+^ for a given dose. Accordingly, renal function and clinical context should inform monitoring targets (e.g., reflexes, respiration, urine output) and, where available, selective use of iMg^2+^ measurement (Magee et al., 2022b; Adomako and Yu, 2024).

### 3.4 Conceptual debate: single pathway vs. multi-pathway synergy

One “single dominant pathway” view holds that NMDA antagonism sufficiently explains MgSO_4_’s antiseizure efficacy by curbing glutamate-mediated excitotoxicity and neuronal synchrony (Magee et al., 2022b; Wilcox et al., 2022). In contrast, a “multipathway synergy” model proposes that combined NMDA blockade, voltage-gated calcium channel modulation, endothelial stabilization, and cerebrovascular effects jointly raise seizure threshold, smooth perfusion, and reduce edema—more consistent with the multisystem pathobiology of preeclampsia and eclampsia (National Institute for Health and Car e Excellence NICE, 2015; Magee et al., 2022b). Current consensus favors a multimechanistic framework, acknowledging that the relative contribution of each pathway likely varies by disease stage, GA, and concomitant therapies. Ongoing work integrating electrophysiology, imaging, and vascular biomarkers may refine these relative weights (National Institute for Health and Car e Excellence NICE, 2015; Magee et al., 2022b; Wilcox et al., 2022).

**FIGURE 1:**
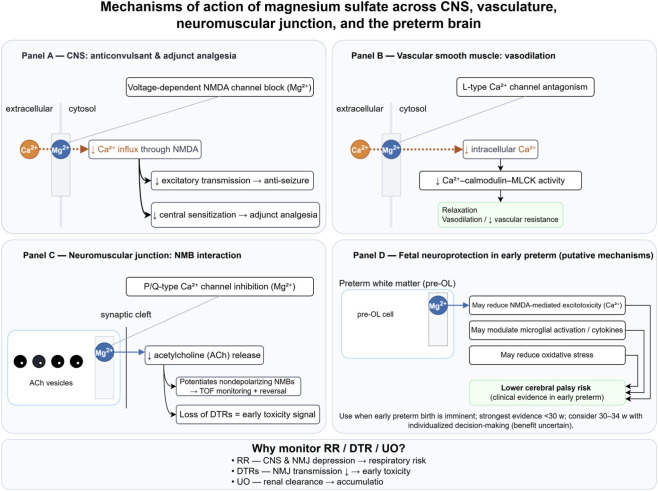
Mechanisms of action of magnesium sulfate across the CNS, vasculature, neuromuscular junction, and the preterm brain. **(A)** Mg^2+^ produces a voltage-dependent block of NMDA receptors, decreasing Ca^2+^ influx and thereby lowering excitatory transmission (anti-seizure) and central sensitization (adjunct analgesia). Panel **(B)** In vascular smooth muscle, antagonism of L-type Ca^2+^ channels reduces intracellular Ca^2+^ and Ca^2+^–calmodulin–MLCK signaling, leading to relaxation and vasodilation. Panel **(C)** At the neuromuscular junction, inhibition of presynaptic P/Q-type Ca^2+^ channels decreases acetylcholine (ACh) release, which potentiates nondepolarizing neuromuscular blockers; loss of deep tendon reflexes (DTRs) is an early clinical signal of toxicity. Panel **(D)** In the preterm brain, Mg^2+^ is hypothesized to mitigate NMDA-mediated excitotoxicity and may modulate microglial activation and oxidative stress; clinically, antenatal MgSO_4_ exposure has been associated with lower risk of cerebral palsy and improved white-matter integrity. Bottom box: Rationale for monitoring respiratory rate (RR), DTRs, and urine output (UO) at the bedside. Color/line conventions: blue arrows, Mg^2+^ effects; orange dotted lines, Ca^2+^ flux; black solid arrows, causal relations; gray thin lines, visual pointers. Schematic only—qualitative pathways are shown; magnitudes are not to scale and no dosing is implied. Abbreviations: ACh, acetylcholine; CNS, central nervous system; DTRs, deep tendon reflexes; MLCK, myosin light-chain kinase; NMB(s), neuromuscular blocker(s); NMDA, N-methyl-D-aspartate; pre-OL, pre-oligodendrocyte; RR, respiratory rate; TOF, train-of-four; UO, urine output.

Figure 1 schematically integrates MgSO_4_ actions on the central nervous system, vasculature, neuromuscular junction, and the preterm brain with bedside monitoring implications.

## 4 Pharmacokinetics, pharmacodynamics, and exposure targets

### 4.1 Key pharmacokinetic (PK) parameters

MgSO_4_ is distributed primarily within the extracellular space, with renal excretion as the dominant elimination pathway; impaired glomerular filtration markedly reduces clearance and prolongs exposure (Adomako and Yu, 2024). Contemporary population PK (popPK) work in women with preeclampsia modeled MgSO_4_ with an apparent clearance of ∼2.98 L/h and a volume of distribution of ∼25.1 L, consistent with mainly extracellular distribution and the clinically observed accumulation when renal function declines (Deng et al., 2024). After IV loading, anticonvulsant effects are rapid; intramuscular (IM) absorption is slower and more variable, with clinical references noting near-immediate onset for IV dosing *versus* delayed and less predictable uptake after IM administration (Hicks and Tyagi, 2023). In an IM-focused PK study in preeclampsia, fewer than two-thirds of participants achieved serum Mg^2+^ ≥2.0 mmol/L by ∼12 h, underscoring the variability of IM regimens (Brookfield et al., 2021).

Pregnancy-specific kinetics interact with these parameters. Expanded plasma volume and increased GFR in normal gestation alter distribution and renal handling, while preeclampsia and its therapies introduce additional heterogeneity (Dimitriadis et al., 2023). Clinically, steady exposure under continuous infusion reflects the balance between distribution and renal elimination; diminished GFR (acute kidney injury or chronic impairment) shifts this balance toward accumulation and elevated toxicity risk (Adomako and Yu, 2024).

### 4.2 Influence of gestational physiology, obesity, and renal impairment

Gestation-associated increases in plasma volume and GFR affect both volume of distribution and clearance; these physiologic shifts, together with disease severity, help explain the interindividual variability seen with standardized dosing in preeclampsia (Dimitriadis et al., 2023). In a 2024 popPK analysis from a Chinese preeclampsia cohort, creatinine clearance (CrCl), body mass index (BMI), and concurrent furosemide significantly influenced drug disposition: higher CrCl and BMI were associated with lower concentrations at a given dose, and diuretic use modified maintenance requirements (Deng et al., 2024). Earlier modeling similarly identified body weight and renal function as major covariates, with higher body weight increasing volume of distribution and effectively reducing the elimination rate under fixed dosing (da Costa et al., 2020). Consistent with these findings, a RCTs in women with obesity (BMI ≥35 kg/m^2^) showed that an “alternate” regimen with a higher maintenance infusion improved attainment of therapeutic levels compared with a standard regimen, supporting weight-informed dose selection in high-BMI populations (Brookfield et al., 2020).

Renal impairment magnifies risk. Because magnesium is cleared renally, reductions in GFR lead to disproportionate increases in serum levels under typical prophylactic infusions; hypermagnesemia with loss of deep-tendon reflexes (DTRs), respiratory depression, and conduction abnormalities can ensue (Adomako and Yu, 2024). Severe iatrogenic hypermagnesemia—most often occurring in the context of renal dysfunction—should prompt immediate cessation of MgSO_4_, IV calcium to antagonize neuromuscular and cardiac effects, aggressive hydration as appropriate, and hemodialysis for refractory or life-threatening toxicity. Contemporary case reports document rapid clinical recovery with dialysis (Si et al., 2024; Miri et al., 2025).

**FIGURE 2:**
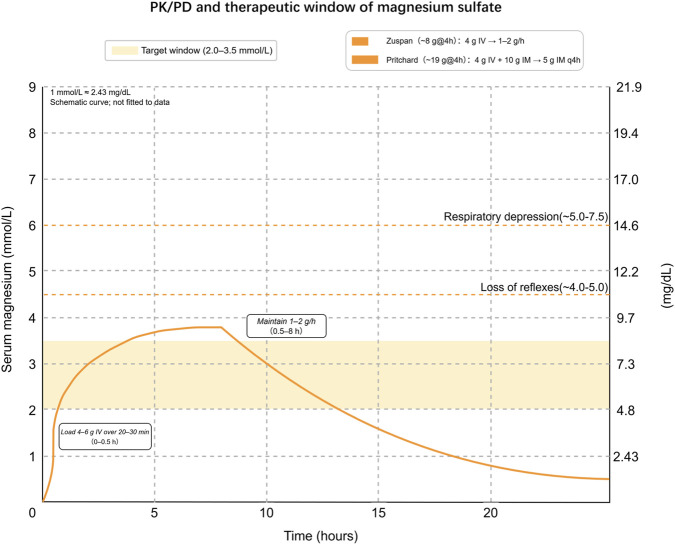
PK/PD and therapeutic window of magnesium sulfate. Schematic concentration–time profile after an IV loading dose followed by maintenance infusion, highlighting the target therapeutic window (2.0–3.5 mmol/L; 4.9–8.5 mg/dL). Approximate toxicity thresholds are shown as dashed bands: loss of deep tendon reflexes ∼4.0–5.0 mmol/L and respiratory depression ∼5.0–7.5 mmol/L. Right axis provides mg/dL (1 mmol/L ≈ 2.43 mg/dL). Example clinical regimens include Zuspan (4 g IV over 20–30 min, then 1–2 g/h) and Pritchard (4 g IV + 10 g IM load [5 g each buttock], then 5 g IM every 4 h). Exposure shifts: reduced renal function → ↑ exposure; larger body size under fixed dosing → ↓ exposure; loop diuretics → ↓ exposure. The curve is schematic (not fitted to data); clinical signs and monitoring (respiratory rate, deep tendon reflexes, urine output, and serum magnesium where available) should guide titration. Sources: therapeutic window and dosing from contemporary obstetric hypertension guidance SOMANZ 2023 (Chen et al., 2023); CMQCC 2022 (Shaheen et al., 2024); toxicity thresholds from a clinical reference on hypermagnesemia (Hellström et al., 2023). **Abbreviations:** PK/PD, pharmacokinetics/pharmacodynamics; MgSO_4_, magnesium sulfate; IV, intravenous; IM, intramuscular; mmol/L, millimoles per liter; mg/dL, milligrams per deciliter; q4h, every 4 h; h, hours; SOMANZ, Society of Obstetric Medicine of Australia and New Zealand; CMQCC, California Maternal Quality Care Collaborative.

Figure 2 illustrates the concentration–time profile, therapeutic window (2.0–3.5 mmol/L), and toxicity thresholds, including contextual shifts in exposure.

### 4.3 Population PK (2015–2025) and simulation-based dosing

Recent popPK studies in preeclampsia generally favor parsimonious structural models—often one-compartment with first-order elimination—capturing substantial between-subject variability *via* covariates such as weight/BMI, CrCl/serum creatinine, and diuretic exposure (Deng et al., 2024; da Costa et al., 2020). In a recent Chinese population PK model for preeclampsia, Monte Carlo simulations suggested that to maintain 2.0–3.5 mmol/L (≈4.9–8.5 mg/dL), a 5 g IV loading dose followed by up to ∼10 g/day maintenance was often required in patients not receiving furosemide, whereas lower maintenance doses (∼2.5 g/day) sufficed when furosemide reduced levels through diuresis; these proposals were explicitly tied to simulated target-attainment probabilities (Deng et al., 2024). The obesity-focused RCT complements the model outputs by demonstrating improved pharmacodynamic target attainment with higher maintenance rates in high-BMI patients on standard obstetric regimens (Brookfield et al., 2020). Collectively, these data support model-informed dose adjustments in patients with extremes of body weight and altered renal function, while recognizing that bedside toxicity monitoring remains the final safety backstop.

### 4.4 Exposure–response and exposure–toxicity

Across obstetric trials, PK studies, and guideline summaries, total serum magnesium concentrations of approximately 2.0–3.5 mmol/L (≈4.9–8.5 mg/dL; 1 mmol/L ≈ 2.43 mg/dL) are commonly cited as the therapeutic range for seizure prophylaxis and treatment in eclampsia and severe preeclampsia (Hicks and Tyagi, 2023; Rosanoff et al., 2022). Within this window, most patients achieve reliable anticonvulsant and neuroprotective effects with an acceptable safety profile, a conclusion echoed across obstetric guidance and recent reviews (De Oliveira et al., 2024; National Institute for Health and Care Excellence NICE, 2019).

As total magnesium rises above the therapeutic range, toxicity tends to follow a predictable clinical sequence. Loss of DTRs is typically reported at concentrations around 4.0–5.0 mmol/L (≈9.7–12.2 mg/dL), followed by progressive respiratory depression at roughly 5.0–7.5 mmol/L (≈12–18 mg/dL), with atrioventricular conduction block and cardiac arrest described at levels exceeding 12.5–15.0 mmol/L (≈30–36 mg/dL) (Hicks and Tyagi, 2023; Rosanoff et al., 2022). These numeric thresholds vary between reports because total serum magnesium includes protein-bound and complexed fractions, whereas only about one-half to two-thirds circulates as ionized, physiologically active Mg^2+^; shifts in pH and albumin can therefore alter the relationship between total and effect-site exposure (Adomako and Yu, 2024). In routine practice, ionized magnesium assays remain limited, so total magnesium is used as a surrogate marker and interpreted alongside bedside signs (respiratory rate, DTRs, urine output) rather than as a stand-alone determinant of safety or toxicity.

### 4.5 Monitoring strategies: clinical signs and serum levels

Most obstetric guidelines and good-practice statements place bedside clinical examination at the center of MgSO_4_ safety monitoring. Serial assessment of DTRs, respiratory rate, and urine output is recommended as the primary safeguard against toxicity, with serum magnesium levels obtained selectively rather than on a fixed schedule (National Institute for Health and Care Excellence (NICE), 2019; Shennan et al., 2021; The ObG Project, 2023). This “clinical-first” approach aligns with the exposure–toxicity sequence summarized in Section 4.4 and reflects the reality that neuromuscular and respiratory changes often precede the availability of laboratory results. In hemodynamically stable patients with preserved renal function receiving standard obstetric regimens, several consensus summaries explicitly state that routine levels are not required (National Institute for Health and Care Excellence (NICE), 2019; Shennan et al., 2021; The ObG Project, 2023).

Serum magnesium measurements play a complementary, targeted role in higher-risk or more complex situations. Because PK variability in preeclampsia, obesity, diuretic use, and renal dysfunction can shift exposure at a given dose (Deng et al., 2024; Hicks and Tyagi, 2023; De Oliveira et al., 2024), levels are useful when: (i) renal function is impaired or deteriorating; (ii) BMI is extreme or dosing deviates from standard protocols; (iii) clinical signs are equivocal or DTRs are unreliable; or (iv) co-therapies plausibly alter magnesium handling (Deng et al., 2024; Adomako and Yu, 2024; Hicks and Tyagi, 2023; National Institute for Health and Care Excellence (NICE), 2019; Shennan et al., 2021; The ObG Project, 2023). In these scenarios, total serum magnesium is interpreted alongside bedside findings to confirm extremes of exposure and guide dose adjustment or interruption, rather than replace clinical monitoring. A more detailed discussion of the “clinical signs *versus* routine levels” debate is provided in Section 8.1, and practice-ready action steps are summarized in Box 1 (Practice checklist).

## 5 Clinical evidence and practice across indications

### 5.1 Preeclampsia and eclampsia

#### 5.1.1 Landmark RCTs and effect size

MgSO_4_ became first-line therapy for the prevention and treatment of eclamptic seizures on the basis of large multicenter randomized trials showing an approximately 50% relative reduction in recurrent seizures *versus* placebo or alternative anticonvulsants, with a favorable maternal outcome signal (Magnesium Sulphate for Prevention of Eclampsia trial, >10,000 participants) (Altman et al., 2002). Head-to-head trials and authoritative syntheses consistently position MgSO_4_ as superior to diazepam or phenytoin for seizure control and maternal outcomes in preeclampsia/eclampsia, cementing its role as the standard of care (Altman et al., 2002; Diaz et al., 2023). Contemporary guideline frameworks (e.g., National Institute for Health and Care Excellence) continue to recommend MgSO_4_ for women with eclampsia or preeclampsia with severe features, reflecting this evidence base (National Institute for Health and Care Excellence NICE, 2019). The World Health Organization likewise designates MgSO_4_ as the primary anticonvulsant for eclampsia worldwide (World Health Organization, 2025).

#### 5.1.2 Regimen families: pritchard vs. Zuspan vs. local modifications

Two regimen “families” dominate practice: Pritchard (IV loading followed by IM maintenance) and Zuspan (IV loading followed by continuous IV infusion at ∼1–2 g/h) (Diaz et al., 2023). Systematic comparisons indicate similar effectiveness for seizure prevention, with differences driven largely by route-specific trade-offs (injection-site pain/abscess for IM regimens *versus* pump/line requirements for continuous IV infusions) (Diaz et al., 2023). Recent reviews and guidelines describe context-specific adaptations—including 12-h vs. 24-h maintenance, reduced-dose (“low-dose”) programs, and IM-first strategies in pump-limited settings—implemented to balance efficacy, toxicity, and feasibility without compromising anticonvulsant protection (National Institute for Health and Care Excellence NICE, 2019; Diaz et al., 2023). A 2024 evidence synthesis reported no clear increase in seizure recurrence with 12-h *versus* 24-h maintenance in selected populations, suggesting that duration can be tailored when careful monitoring is ensured (Shaheen et al., 2024).

To operationalize regimen choice across settings, Table 1 contrasts the Pritchard (IM-based), Zuspan (IV infusion), and common low-dose/hybrid variants by dosing, equipment dependence, advantages, limitations, and bedside safety “hold” criteria (reflexes, respiratory rate, urine output).

**TABLE 1:** Magnesium sulfate regimen families for preeclampsia/eclampsia.

Regimen (family)	Loading (IV/IM)	Maintenance	Route and equipment	Pros (when to prefer)	Limitations/Common AEs	Fit-for-setting (examples)	Monitoring guardrails
Pritchard (IM-based)	4 g IV over 5–10 min **plus** 10 g IM total (5 g into each buttock). Use 50% MgSO_4_ for IM; optional 1 mL 2% lidocaine per buttock.	5 g IM q4h (alternating buttocks) for 24 h after last seizure or delivery **if** reflexes present, RR ≥12/min, and UO ≥25–30 mL/h.	IV + deep IM injections; no infusion pump required; trained staff for IM technique.	Works without infusion pumps; robust where continuous IV infusion is unavailable; effective seizure control with large evidence base.	Painful IM injections; injection-site hematoma/abscess; slower titration; caution with bleeding risk or severe thrombocytopenia; toxicity risk without close monitoring.	Low-resource wards; during transport/transfer; limited pump supply; fallback when pumps fail.	Hold dose if patellar reflex absent, RR <12/min, or UO <25–30 mL/h (≈<100 mL/4 h). Keep calcium gluconate 10% 10 mL IV available for toxicity.
Zuspan (IV infusion)	4 g IV over 10–20 min (diluted in 100–200 mL NS/D5). If recurrent seizure, give an additional 2–4 g IV over ∼5 min.	1–2 g/h continuous IV infusion (commonly 1 g/h) for 24 h after last seizure or delivery; adjust per clinical status and renal function.	Continuous IV infusion *via* infusion pump with reliable IV access.	Easier titration; fewer IM complications; better patient comfort; rapid IV bolus possible if seizures recur.	Requires pump, power, and consumables; infusion errors possible; IV line maintenance needed; caution with renal impairment (accumulation).	Higher-resource labor wards/ICU/OR with pumps; when frequent reassessment and titration are feasible.	As above: hold/slow infusion if reflexes absent, RR <12/min, or oliguria; consider serum Mg if renal dysfunction or toxicity suspected.
Low-dose/Hybrid (e.g., Dhaka; Jana/Burdwan variants)	Typical Dhaka: 4 g IV over 10–15 min **plus** 6 g IM total (3 g each buttock) = 10 g. Some variants: 3 g IV + 5 g IM = 8 g total.	2.5 g IM q4h for 24 h after last seizure/delivery; 2 g IV re-bolus for recurrent seizure per local protocol.	IV + lower IM doses; designed to reduce total MgSO_4_ exposure when monitoring capacity is limited.	Lower cumulative dose; may reduce toxicity where BMI is low and monitoring is limited; used in some South Asian programs.	Evidence base mixed and context-specific; not universally endorsed; still requires IM injections; ensure clear escalation triggers.	Centers adopting low-dose protocols based on local audits/trials and lower average BMI; programs aiming to reduce dose while preserving efficacy.	Same clinical monitoring; define explicit escalation plan for recurrent seizures and for transfer.

Doses refer to grams of magnesium sulfate (MgSO_4_), not elemental magnesium. 50% = 500 mg/mL; 20% = 200 mg/mL. Example: 4 g = 8 mL (50%) or 20 mL (20%).

Standard hold/toxicity criteria: absent patellar reflexes; RR <12/min (some checklists use <16/min); UO <25–30 mL/h (≈<100 mL/4 h). Keep 10 mL of 10% calcium gluconate IV available for toxicity reversal.

Do not delay indicated delivery to complete MgSO_4_; continue for 12–24 h postpartum as per protocol.

Abbreviations: **AE**, adverse event; **D5**, 5% dextrose; **IM**, intramuscular; **IV**, intravenous; **ICU**, intensive care unit; **NS**, normal saline; **OR**, operating room; **RR**, respiratory rate; **UO**, urine output.

Source note; NICE NG133 (National Institute for Health and Care Excellence NICE, 2019); SOMANZ (2023) (Society of Obstetric Medicine of Austr alia and New Zealand SOMANZ, 2023); The Dhaka low-dose protocol derives from Begum et al. (2001); Dosing and monitoring synthesized from AHRQ Comparative Effectiveness Review (2023) table of commonly used MgSO_4_ regimens (Steele et al., 2023); ACOG Practice Bulletin No.222 (2020) (American College of Obstetricians and Gynecologists, 2020); and WHO Prequalification summaries (WHOPAR, updated 2022) (World Health Organization, 2020); See references for details.

In practice, infusion regimens are preferable where reliable pumps and frequent reassessment are available; IM-based regimens remain pragmatic when pumps are scarce or transport is anticipated; and low-dose variants should be limited to programs with local evidence and explicit escalation pathways.

#### 5.1.3 Regimen and route choices in resource-constrained settings

Debate continues over how best to operationalize MgSO_4_ regimens where infusion pumps and continuous monitoring are limited, but several practical themes are consistent. Contemporary systematic evidence suggests that fixed-rate IV infusions provide steadier target attainment than serial IV boluses, whereas bolus-dominant approaches may be pragmatic when trained staff can perform frequent clinical assessments and re-dosing in settings without reliable pumps (Diaz et al., 2023). Route feasibility also matters: IM-based programs avoid pump dependence but carry risks of injection-site pain and local complications, whereas IV infusions require dependable pumps or gravity-drip systems, uninterrupted drug and line supply, and sufficient bedside monitoring capacity (Diaz et al., 2023).

Qualitative work from low- and middle-income countries highlights that, despite proven benefit, MgSO_4_ remains underused because of supply-chain problems, fears of toxicity in the absence of laboratory support, and gaps in staffing and training for monitoring reflexes, respirations, and urine output (Eddy et al., 2022). Global guidance therefore emphasizes selecting a regimen that is realistic for local resources while preserving effective dosing and basic safety checks—prioritizing bedside clinical signs as the primary safeguard and reserving serum magnesium levels for renal impairment or atypical courses (World Health Organization, 2025; National Institute for Health and Care Excellence NICE, 2019). Broader debates about bolus-versus-infusion “dominance” and regimen simplification in constrained settings are revisited in Section 8.2, and consolidated regimen and program supports are summarized in Box 1 (Practice checklist).

### 5.2 Fetal neuroprotection

#### 5.2.1 Evidence base: meta-analyses and cohorts

Across randomized trials synthesized in recent updates, antenatal MgSO_4_ given before imminent early preterm birth reduces CP by roughly one-third and lowers the composite of death or CP, with no clear effect on mortality alone. Several reviews also note a probable reduction in severe intraventricular hemorrhage and neutral findings for bronchopulmonary dysplasia/chronic lung disease (Shaheen et al., 2024; Crowther et al., 2023). Recent meta-analyses published alongside the 2024 Cochrane update reaffirm these patterns, emphasizing consistent CP reduction with limited data on longer-term neurodevelopment beyond 2 years (Shaheen et al., 2024; Crowther et al., 2023).

#### 5.2.2 Gestational-age window, dosing (single vs. repeat), neonatal outcomes

International guidance recommends routine use when birth before approximately 30–32 weeks’ gestation is imminent, with the strongest recommendation at <30 weeks and more individualized decisions between 30 and 34 weeks depending on timing and likelihood of delivery (Shennan et al., 2021; Hywel Dda University Health Board, 2024). In the MAGENTA randomized clinical trial of antenatal MgSO_4_ at 30–34 weeks’ gestation, MgSO_4_ was administered as a loading dose only (without a maintenance infusion) and did not significantly reduce death or CP at 2 years, tempering expectations for benefit at later gestations and supporting the emphasis on earlier GA (Crowther et al., 2023). This result should be interpreted cautiously, as the lack of observed benefit at 30–34 weeks may reflect later-gestation injury biology and/or the shorter exposure achieved with a bolus-only strategy, and it does not exclude the possibility that standard loading-plus-maintenance regimens could yield different effects in selected later-gestation contexts (Crowther et al., 2023). Typical neuroprotection regimens comprise a 4-g IV loading dose followed by a 1 g/h infusion until birth or up to 24 h, though operational details vary by health system; for fetal neuroprotection, protocols are generally fixed-dose and do not recommend routine adjustment based on maternal body size or distribution volume (with individualization focused on safety, such as renal impairment and toxicity monitoring) (Hywel Dda University Health Board, 2024; Health Service Executive (HSE), 2013). Repeat dosing is not routine, with some national guidance allowing one repeat course only if ≥ 24 h have elapsed and preterm birth is again imminent (Shennan et al., 2021; Hywel Dda University Health Board, 2024; Health Service Executive HSE, 2013). In line with the evidence base, expected neonatal effects include reduced CP and reduced death/CP composite, a possible reduction in severe intraventricular hemorrhage, no clear mortality difference, and no consistent signal for bronchopulmonary dysplasia/chronic lung disease (Shepherd et al., 2024; Crowther et al., 2023).

**FIGURE 3:**
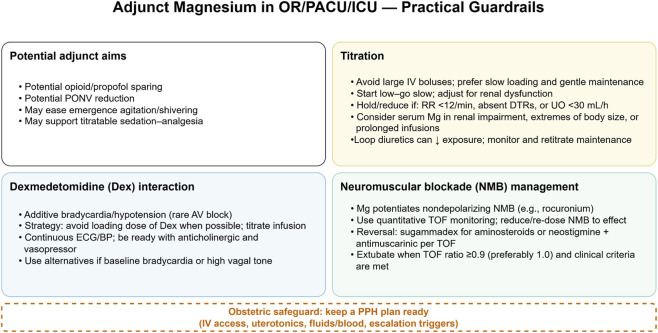
Adjunct magnesium in OR/PACU/ICU—practical guardrails. Potential adjunct aims include potential opioid/propofol sparing, possible PONV reduction, and improved emergence comfort (e.g., agitation/shivering), recognizing that effect sizes are context-dependent. Titration: avoid large boluses; use gentle maintenance and hold/reduce if RR < 12/min, absent DTRs, or UO < 30 mL/h; adjust for renal impairment; obtain levels selectively (e.g., prolonged infusion, extremes of body size, renal dysfunction). Dexmedetomidine interaction: additive bradycardia/hypotension—prefer no loading dose, titrate, and prepare for treatment. NMB management: magnesium potentiates nondepolarizing neuromuscular blockers—use quantitative TOF monitoring and standard reversal; extubate at TOF ≥0.9–1.0 with clinical readiness. Obstetric safeguard: maintain a PPH contingency plan. Abbreviations: OR, operating room; PACU, post-anesthesia care unit; ICU, intensive care unit; PONV, postoperative nausea and vomiting; NMB, neuromuscular blockade; TOF, train-of-four; RR, respiratory rate; DTR, deep tendon reflexes; UO, urine output; MgSO_4_, magnesium sulfate; IV, intravenous.

Figure 3 summarizes the gestational-age–anchored decision tree for antenatal MgSO_4_ and the conditions for repeat dosing without delaying delivery.

#### 5.2.3 Practical dose and timing considerations near delivery

For antenatal neuroprotection, current data do not show a strong dose–response gradient beyond achieving timely exposure with recommended loading and maintenance regimens. Notably, MAGENTA at 30–34 weeks evaluated a loading-dose–only regimen (without a maintenance infusion) and did not significantly reduce death or CP at 2 years; this finding is hypothesis-generating and suggests that bolus-only exposure may be insufficient for neuroprotection at later gestations, reinforcing the importance of regimen design and exposure duration in future trials (Crowther et al., 2023). Contemporary systematic reviews and implementation reports support the use of standardized, guideline-based regimens with robust clinical monitoring, rather than frequent dose escalation or complex individualization, as long as key eligibility and safety criteria are met (Shepherd et al., 2024; Shennan et al., 2021).

With respect to timing, classic teaching aims to start MgSO_4_ approximately 4 h before anticipated birth, but guideline panels explicitly advise administering treatment even when delivery is expected in less than 4 h, because potential benefit appears to persist and deferring urgent delivery solely to extend the infusion window is inappropriate (Shennan et al., 2021; Hywel Dda University Health Board, 2024). Implementation series from national programs report median start-to-birth intervals around 3–4 h in routine practice, underscoring that exact timing is often constrained and supporting a pragmatic “give when birth is likely” stance rather than rigid timing thresholds (Hellström et al., 2023).

Indications, standard regimens, and repeat-dose policies for antenatal MgSO_4_ are summarized in Box 1 (Practice checklist), while remaining uncertainties about dose–response and timing in borderline scenarios are discussed further in Section 8.4.

### 5.3 Perioperative and ICU co-administration

Across operating rooms (ORs), post-anesthesia care units (PACUs), and ICUs, IV MgSO_4_ has reproducible anesthetic- and analgesic-sparing effects attributable to NMDA receptor antagonism and calcium-channel antagonism. A contemporary meta-analysis of spine surgery randomized trials shows lower postoperative opioid consumption at 24 h, reduced intraoperative remifentanil requirements, and a clinically meaningful decrease in postoperative nausea and vomiting, with a trade-off of slightly prolonged orientation and recovery times—placing MgSO_4_ as a reasonable adjunct when early opioid minimization and postoperative nausea and vomiting reduction are priorities (Yue et al., 2022). Evidence outside spine surgery is directionally consistent: in elderly patients undergoing Endoscopic Retrograde Cholangiopancreatography with propofol-based sedation, a single 40 mg/kg MgSO_4_ bolus reduced propofol use by ≈ 21% and lowered respiratory depression and involuntary-movement events without prolonging recovery, supporting a dose-sparing role during titrated hypnotic sedation (Chen et al., 2023). Randomized perioperative data also suggest reduced emergence agitation and lower intraoperative remifentanil requirements when MgSO_4_ is added to general anesthesia, aligning with its central antinociceptive profile (Su et al., 2023).

Drug–drug interactions are central to safe co-administration. With propofol, MgSO_4_ typically lowers the hypnotic requirement; clinicians should anticipate lower doses to achieve comparable sedation targets and monitor for delayed awakening when high total hypnotic/adjunct loads accrue (Yue et al., 2022; Chen et al., 2023). When combined with dexmedetomidine, hemodynamic depressant effects may be additive, because dexmedetomidine independently increases intraoperative hypotension and bradycardia—particularly when a loading dose is used—warranting cautious titration and readiness to treat bradycardia and vasodilatory hypotension (Wang et al., 2024). Co-administration with benzodiazepines and opioids can further reduce opioid need *via* NMDA antagonism, but practitioners should watch for enhanced central nervous system depression and slower emergence in susceptible patients, especially older adults and those receiving multiple sedatives (Yue et al., 2022; Chen et al., 2023).

Potentiation of nondepolarizing neuromuscular blockers (NMBs)—notably rocuronium—remains a practical concern because Mg^2+^ decreases presynaptic acetylcholine release and postsynaptic excitability. Accordingly, the 2023 American Society of Anesthesiologists guideline recommends quantitative neuromuscular monitoring and pharmacologic reversal guided by objective train-of-four metrics to avoid residual paralysis in any setting where NMBs are used and potentiation is possible (Thilen et al., 2023).

In obstetric anesthesia, benefit–risk evaluation must also consider uterine tone. A 2021 meta-analysis found no significant increase in uterine atony or postpartum hemorrhage among women receiving MgSO_4_ *versus* those not receiving MgSO_4_, tempering concerns that adjunct use inevitably worsens uterine contractility; nonetheless, vigilance for hypotension and careful titration around delivery remain good practice (Pergialiotis et al., 2021).

**FIGURE 4:**
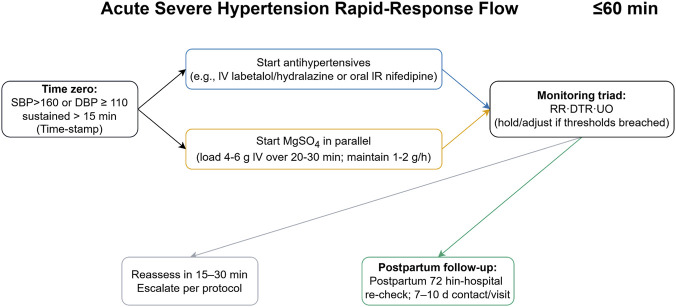
Acute severe hypertension rapid-response flow. Time-stamp severe BP (SBP ≥160 or DBP ≥110 for ≥15 min), initiate antihypertensives and MgSO4 in parallel within 30–60 min, apply monitoring triad (RR/DTR/UO), reassess in 15–30 min and escalate per protocol, and complete postpartum follow-up (72-h in-hospital re-check and 7–10-day contact/visit). Abbreviations: RR, respiratory rate; DTR, deep tendon reflexes; UO, urine output; IR, immediate-release.

Figure 4 consolidates practical guardrails for adjunct MgSO_4_ use in the OR/PACU/ICU—goals, titration with bedside safety thresholds, dexmedetomidine interaction, quantitative NMB monitoring and reversal, and obstetric hemorrhage safeguards.

For perioperative/ICU adjunct use—titration, interactions, NMB monitoring, and obstetric precautions—see Box 1 (Practice checklist).

### 5.4 Hypertensive emergencies in pregnancy

#### 5.4.1 Combination therapy with MgSO_4_ and antihypertensives

**FIGURE 5:**
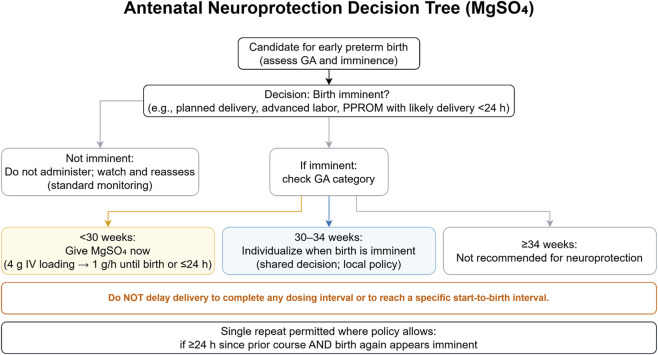
Antenatal Neuroprotection Decision Tree (MgSO_4_). Start when birth is imminent: <30 weeks favored; 30–34 weeks individualized per local policy; ≥34 weeks not recommended. Do not delay delivery to complete a dosing interval or to reach a specific start-to-birth interval. A single repeat may be considered where permitted if ≥24 h since prior course and preterm birth again appears imminent. GA, gestational age; MgSO_4_, magnesium sulfate; IV, intravenous; PPROM, preterm prelabor rupture of membranes; h, hours.

In acute-onset severe hypertension (systolic ≥160 mmHg or diastolic ≥110 mmHg), MgSO_4_is indicated for seizure prophylaxis or treatment, while blood pressure (BP) should be lowered urgently with fast-acting agents such as IV labetalol, oral immediate-release nifedipine, IV hydralazine, or IV nicardipine. IV nitroglycerin is appropriate when pulmonary edema complicates preeclampsia (Countouris et al., 2025). Convergent guidance (e.g., National Institute for Health and Care Excellence) endorses this combined approach and agent set, with local protocolization recommended so that timely treatment is delivered consistently across labor and delivery (L&D), emergency departments, and ICU settings (National Institute for Health and Care Excellence NICE, 2019). Where regionally available, urapidil appears in contemporary summaries as an IV option within structured algorithms, although evidence and availability are heterogeneous (Bhat et al., 2023). Figure 5 summarizes the core clinical elements of this response—parallel initiation of antihypertensives and MgSO_4_ and early reassessment—while the design of order sets and other implementation tools is discussed in Section 7.

#### 5.4.2 Time-to-target BP as a pharmacodynamic endpoint

Quality-improvement programs and clinical studies commonly track time to BP control (e.g., achieving <150/100 or <160/110 mmHg within 30–60 min) as a pragmatic pharmacodynamic endpoint that is tightly linked to maternal stroke risk and adherence to recommended therapy (Countouris et al., 2025; California Maternal Quality Care Collaborative CMQCC, 2022). Recent multicenter materials from initiatives such as Alliance for Innovation on Maternal Health (AIM) and California Maternal Quality Care Collaborative specify treatment within 30–60 min of a qualifying BP as a key process measure (California Maternal Quality Care Collaborative CMQCC, 2022). These operational benchmarks align with guideline emphasis on rapid therapy and provide a shared language for comparing practice across low- and high-resource settings (National Institute for Health and Care Excellence NICE, 2019; Countouris et al., 2025; California Maternal Quality Care Collaborative CMQCC, 2022). The practical challenge of embedding these targets into workflows—through order sets, alerting, and data feedback—is addressed in Section 7.

#### 5.4.3 Comparative effectiveness signals and practicality

Comparative studies suggest that oral immediate-release nifedipine often achieves faster BP reduction than IV labetalol or hydralazine, with similar short-term safety, supporting its use when IV access is delayed or not required for other reasons (S et al., 2023). Conversely, IV labetalol remains attractive when continuous monitoring, concurrent IV therapies, or rate titration are needed, while IV hydralazine or nicardipine provide alternatives when β-blockade is contraindicated or insufficient (National Institute for Health and Care Excellence NICE, 2019). Agent selection should incorporate comorbidity and logistics: nitroglycerin for pulmonary edema, avoidance of β-blockers in bradycardia or asthma, and the practicalities of ward *versus* Emergency Department/L&D environments (National Institute for Health and Care Excellence NICE, 2019; Countouris et al., 2025). A practical takeaway is to choose a locally reliable protocol—with clear drug choice, dosing, and escalation—that reliably achieves the desired time-to-target BP while co-administering MgSO_4_ when indicated and maintaining bedside monitoring to prevent hypotension and overtreatment (National Institute for Health and Care Excellence NICE, 2019; Countouris et al., 2025; California Maternal Quality Care Collaborative CMQCC, 2022; S et al., 2023).

## 6 Safety, toxicity, and monitoring

MgSO_4_ is highly effective when used with appropriate monitoring, yet its therapeutic window is narrow and exposure varies with renal function and body size (Adomako and Yu, 2024; Hywel Dda University Health Board, 2024). This section synthesizes adverse effects, toxicity thresholds and reversal, fetal/neonatal considerations (including lactation), monitoring strategies, and dose–safety issues in renal impairment and extreme BMI, with practice-ready takeaways.

### 6.1 Adverse-effect spectrum (maternal vs. neonatal)

Maternal adverse effects are usually mild and dose related—flushing, warmth, nausea, vomiting, lethargy, dizziness—and rarely require discontinuation when infusions are titrated and clinical signs are monitored (Shepherd et al., 2024; Brookfield and Mbata, 2023). More serious events include hypotension, respiratory depression or arrest, atrioventricular conduction block, and cardiac arrest, typically in the setting of excessive serum magnesium (e.g., reduced glomerular filtration) or concurrent sedatives/neuromuscular blockers (Adomako and Yu, 2024; Magley et al., 2025; Merck & Co. and Inc, 2025). In randomized trials and systematic reviews of antenatal neuroprotection, serious maternal adverse events were uncommon and generally manageable with protocolized monitoring (Shepherd et al., 2024).

Neonatal effects after *in utero* exposure near delivery may include transient hypotonia, lower Apgar scores, or short-term NICU admission signals in some cohorts; however, these findings must be interpreted against consistent reductions in CP and the composite of death or CP among very preterm infants exposed to antenatal MgSO_4_, without a clear adverse mortality signal (Shepherd et al., 2024). When high cumulative maternal dosing is given very close to delivery, nursery observation for tone and respiration is prudent (Shepherd et al., 2024; Brookfield and Mbata, 2023).

### 6.2 Toxicity thresholds and reversal

As detailed in Section 4.4, magnesium toxicity follows a concentration-dependent clinical sequence from loss of DTRs to progressive respiratory depression and, at extreme exposures, atrioventricular block and cardiac arrest, as summarized in pharmacology references and case series (Adomako and Yu, 2024; Merck & Co. and Inc, 2025). In practice, any significant neuromuscular or respiratory compromise in a patient receiving MgSO_4_ should be treated as toxicity regardless of the measured level, with clinical signs guiding urgency and escalation.

Management begins with immediate discontinuation of MgSO_4_ and prompt administration of IV calcium, typically 10 mL of 10% calcium gluconate (≈1 g) given slowly IV, to counteract neuromuscular and cardiac effects (Adomako and Yu, 2024; Magley et al., 2025; Merck & Co. and Inc, 2025). Supportive care includes airway protection and assisted ventilation as needed, isotonic fluids and loop diuretics when appropriate, and continuous cardiac and respiratory monitoring (Adomako and Yu, 2024; Magley et al., 2025; Merck & Co. and Inc, 2025). In severe or refractory hypermagnesemia—particularly in the setting of renal failure—hemodialysis or continuous renal replacement therapy provides enhanced elimination and should be considered early when clinical deterioration persists despite initial measures (Adomako and Yu, 2024; Magley et al., 2025).

### 6.3 Fetal/neonatal safety and lactation

Antenatal MgSO_4_ for neuroprotection reduces CP and the composite of death/CP among early preterm births without a clear increase in neonatal mortality, and with low rates of serious maternal complications under protocolized care (Shepherd et al., 2024). Neonatal hypotonia or respiratory depression may occur after high maternal doses given close to delivery but is generally transient and manageable with supportive care (Shepherd et al., 2024). During breastfeeding, transfer into milk is minimal; the estimated relative infant dose is very low, and clinically important effects in breastfed term infants are unlikely, although monitoring is reasonable if the maternal infusion is ongoing immediately postpartum [Drugs and Lactation Database (LactMed®), 2006]. Drugs and Lactation Database and clinical reviews consider MgSO_4_ compatible with breastfeeding [Brookfield and Mbata, 2023; Drugs and Lactation Database (LactMed®), 2006)].

### 6.4 Practical monitoring protocol—who needs levels?

Building on the exposure–toxicity relationships in Section 4.4 and the monitoring debate discussed in Section 8.1, current guidance supports a clinical-first, level-supported strategy for patients receiving MgSO_4_. All patients should undergo baseline assessment of mental status, respiratory rate, DTRs, and urine output, followed by serial respiratory rate/deep tendon reflexes/urine output.

(RR/DTR/UO) checks at the bedside to prevent unrecognized accumulation and respiratory compromise (World Health Organization, 2025; Magee et al., 2022a; Society of Obstetric Medicine of Austr alia and New Zealand SOMANZ, 2023). In hemodynamically stable patients with preserved renal function on standard obstetric regimens, many consensus statements indicate that routine serum magnesium levels are not required when examinations are reliable (World Health Organization, 2025; Magee et al., 2022a; Society of Obstetric Medicine of Austr alia and New Zealand SOMANZ, 2023).

Serum magnesium measurements are reserved for situations in which exposure is likely to be shifted or clinical signs are less trustworthy. Guidelines recommend selective levels in the presence of renal impairment or deterioration, prolonged infusions, extremes of body size or atypical dosing, and equivocal or confounded examinations (e.g., absent or unreliable reflexes) (World Health Organization, 2025; Magee et al., 2022a; Brookfield and Mbata, 2023; Society of Obstetric Medicine of Austr alia and New Zealand SOMANZ, 2023). Where ionized magnesium (iMg^2+^) assays are unavailable, total serum magnesium remains the practical laboratory metric and is interpreted alongside bedside findings rather than in isolation (Adomako and Yu, 2024).

In anesthetic and critical-care settings, concurrent sedatives and NMBs increase the risk of apnea and residual paralysis. Professional guidance therefore recommends quantitative neuromuscular monitoring and agent-specific reversal to mitigate MgSO_4_-related potentiation of NMBs, with the same principles of clinical-first monitoring and targeted levels applied in this higher-risk context (Thilen et al., 2023; Rodney et al., 2024).

### 6.5 Renal impairment and extreme BMI: practical dosing and monitoring

As discussed in Section 8.1, dosing and monitoring in renal impairment and extreme BMI remain areas of active debate. From a practical standpoint, several principles are clear. Because magnesium is predominantly renally cleared, reduced glomerular filtration rate (acute kidney injury or chronic kidney disease) increases exposure at any given infusion rate and raises the risk of DTR loss, respiratory depression, and conduction defects (Adomako and Yu, 2024; Magley et al., 2025). In such patients, guidelines and reviews advise lower maintenance doses, cautious titration, and a lower threshold for checking levels or interrupting therapy when clinical signs become unreliable (World Health Organization, 2025; Magee et al., 2022a; Brookfield and Mbata, 2023; Society of Obstetric Medicine of Austr alia and New Zealand SOMANZ, 2023). The goal is to keep exposure within the therapeutic range outlined in Section 4.4 while avoiding abrupt toxicity.

By contrast, obesity increases the apparent volume of distribution and can blunt concentrations achieved by fixed infusions. PopPK analyses in preeclampsia identify BMI/weight and creatinine clearance as key covariates influencing exposure, and simulations suggest that some individuals may require higher maintenance rates to reach the desired therapeutic range, whereas diuretics and reduced renal function lower requirements (Deng et al., 2024). Clinicians must balance underexposure (breakthrough seizures) against overexposure (toxicity), recognizing that model-informed dosing can complement but not replace bedside monitoring and serial clinical assessment (Magee et al., 2022a). Reviews also highlight that, in carefully selected patients, postpartum magnesium duration may be shortened (e.g., 12 h vs. 24 h) without increasing eclampsia, thereby reducing cumulative exposure, although practice varies and should align with local protocols and individual risk (Quist-Nelson et al., 2024).

In day-to-day care, a pragmatic approach is to maintain standard loading doses, apply modest maintenance adjustments in women with impaired or changing renal function or extreme BMI, and reserve serum magnesium levels for situations in which renal function is compromised, dosing deviates from standard regimens (such as high-BMI adjustments), or the clinical examination is unreliable (World Health Organization, 2025; Deng et al., 2024; Magee et al., 2022a; Society of Obstetric Medicine of Austr alia and New Zealand SOMANZ, 2023). Section 8.1 expands on the underlying controversy around routine levels *versus* targeted monitoring, and Box 1 (Practice checklist) consolidates practical action steps and monitoring thresholds for these higher-risk groups.

## 7 Implementation science and systems-level advances

Having reviewed pharmacology and clinical evidence, we now examine how MgSO_4_ is implemented and governed at the systems level.

### 7.1 Digital order sets, EHR alerts, and automated monitoring

Standardized electronic order sets that pair a MgSO_4_ loading dose with maintenance, embed contraindications, and auto-populate nursing checks (respiratory rate, deep-tendon reflexes, urine output) are a cornerstone of reliable care in hypertensive emergencies of pregnancy. National bundles recommend enterprise-wide build and default escalation steps to reduce omissions and delays (Birth, 2022; Institute for Healthcare Improvement. AIM Patient Safety Bundles, 2025). Best Practice Advisories (BPAs) that fire on sustained severe BP and link directly to the order set can improve timeliness, but effects are heterogeneous and susceptible to alert fatigue unless trigger logic is tight and roles are clearly defined (Institute for Healthcare Improvement. AIM Patient Safety Bundles, 2025; Spencer et al., 2021). In a pre–post evaluation, an automated alert increased the proportion treated within 1 h yet had neutral effects on some downstream elements, underscoring that alerts must be embedded within end-to-end workflows (order sets, handoffs, escalation) rather than deployed in isolation (Spencer et al., 2021). High-performing sites also standardize default timing for laboratory surveillance in renal impairment (selective magnesium levels), document neuromuscular-monitoring plans when nondepolarizing blockers are used, and use auto-generated reassessment tasks at 15–30 min to close loops (Birth, 2022; Institute for Healthcare Improvement. AIM Patient Safety Bundles, 2025).

Digitized maternal early-warning systems Modified Early Warning Score/Modified Early Obstetric Warning System (MEWS/MEOWS) integrate vital signs and symptoms to trigger team responses and open the severe-hypertension pathway; a recent national development/validation provides a template for electronic health record (EHR) implementation with actionable thresholds that can automatically surface MgSO_4_ and antihypertensive orders (Gerry et al., 2024). After discharge, remote postpartum BP programs with algorithm-driven triage detect more hypertension and reduce readmissions, providing a structured pathway for timely antihypertensives and reconsideration of MgSO_4_ when neurologic symptoms or severe features emerge (Mujic et al., 2024; Hauspurg et al., 2024). The 2025 American Heart Association Scientific Statement endorses structured remote monitoring and explicit escalation timelines in the postpartum period (Countouris et al., 2025).

### 7.2 QI bundles and nursing safety checklists

The AIM Severe Hypertension in Pregnancy safety bundle organizes work into Readiness, Recognition/Prevention, Response, and Reporting/Learning, explicitly pairing MgSO_4_ for seizure prophylaxis/treatment with rapid-acting antihypertensives, team roles, and data capture (Birth, 2022). The Institute for Healthcare Improvement/AIM Change Package operationalizes this framework with simulation scripts, standardized handoffs, and bedside nursing checklists that document reflexes, respirations, urine output, and infusion integrity at defined intervals (Institute for Healthcare Improvement. AIM Patient Safety Bundles, 2025). Central to these programs is the process metric “treat severe BP within 30–60 min”—with repeat BP at 15 minutes—tracked *via* simple run charts or statistical process control to drive weekly feedback and course correction (California Maternal Quality Care Collaborative CMQCC, 2022; Institute for Healthcare Improvement. AIM Patient Safety Bundles, 2025; Combs et al., 2022). California Maternal Quality Care Collaborative materials translate the metric into practical steps (who repeats the BP, who orders, who administers the dose) to compress latency in real care environments (California Maternal Quality Care Collaborative CMQCC, 2022).

### 7.3 Real-world adherence and adverse-event reduction

State perinatal quality collaboratives and health systems implementing AIM report increases in the proportion of patients treated within 60 min and concurrent improvements in reliability of MgSO_4_ administration for indicated patients. Several collaboratives also report declines in HDP-related severe maternal morbidity (SMM), though effects vary with baseline performance, staffing, and bundle uptake (Birth, 2022). Neutral or mixed findings are common when sites deploy EHR alerts without parallel work on order-set adoption, role clarity, and bedside checklists, emphasizing the importance of a whole-system approach (Institute for Healthcare Improvement. AIM Patient Safety Bundles, 2025; Spencer et al., 2021). Across programs, equity stratifiers (race/ethnicity, language, rurality) are applied to every key performance indicators (KPIs) so that closing gaps is an explicit objective rather than an incidental outcome—an approach increasingly embedded in AIM and California Maternal Quality Care Collaborative dashboards (California Maternal Quality Care Collaborative (CMQCC), 2022; Birth, 2022; Institute for Healthcare Improvement. AIM Patient Safety Bundles, 2025).

### 7.4 Low-resource adaptations and World Health Organization/International Federation of Gynecology and Obstetrics initiatives

Where pumps and laboratories are limited, feasible MgSO_4_ regimens (e.g., protocolized IV or IM maintenance), simplified observation sheets, and paper-to-digital escalation pathways enable safe implementation at district hospitals (California Maternal Quality Care Collaborative CMQCC, 2022). The WHO Fact Sheet identifies MgSO_4_ underuse as a remediable systems failure and urges national adoption of standardized protocols, supply-chain assurance, and routine audit-and-feedback (California Maternal Quality Care Collaborative CMQCC, 2022). WHO’s QCN provides an implementation architecture—from national coaching to facility-level micro-audits—with tools that can be adapted for MgSO_4_ order sets, bedside checklists, and data flows as sites digitize over time (World Health Organization). These resources offer a pragmatic “on-ramp” for facilities beginning with paper algorithms and gradually advancing to EHR-integrated workflows.

### 7.5 Logic model (one sentence)

Inputs → Activities → Outputs → Outcomes: leadership + stocked kits + EHR builds + training → order sets + BPAs + early-warning systems + simulations + remote BP programs + dashboards → faster initiation, complete monitoring, reliable handoffs → more cases treated in ≤60 min, higher indicated MgSO_4_ use, fewer eclampsia/SMM events, and narrowed equity gaps (Countouris et al., 2025; California Maternal Quality Care Collaborative (CMQCC), 2022; Birth, 2022; Institute for Healthcare Improvement. AIM Patient Safety Bundles, 2025; Gerry et al., 2024; World Health Organization).

### 7.6 Compact KPI panel (define, then act)

#### 7.6.1 Process

(i) % of severe BP episodes treated in ≤60 min (numerator: therapy started within 60 min of sustained severe BP; denominator: qualifying episodes), goal ≥80% and rising (California Maternal Quality Care Collaborative CMQCC, 2022; Institute for Healthcare Improvement. AIM Patient Safety Bundles, 2025; Combs et al., 2022); (ii) % of indicated patients receiving MgSO_4_, goal ≥95% (Birth, 2022); (iii) Bundle adherence score (order-set use, repeat BP at 15 min, documented escalation, checklist completion), goal ≥85% (Institute for Healthcare Improvement. AIM Patient Safety Bundles, 2025).

#### 7.6.2 Outcomes

Eclampsia rate and HDP-related SMM, both trending downward as timeliness improves (Birth, 2022; Combs et al., 2022).

#### 7.6.3 Safety

Mg toxicity events per 1,000 administrations and unplanned ICU transfers, each reviewed with root-cause analysis and countermeasures (Institute for Healthcare Improvement. AIM Patient Safety Bundles, 2025).

#### 7.6.4 Equity

Report all KPIs by race/ethnicity, language, and rurality; trigger a countermeasure if any subgroup lags the aggregate by > 10 percentage points or shows a worsening trend (Birth, 2022; Institute for Healthcare Improvement. AIM Patient Safety Bundles, 2025).

#### 7.6.5 Methods note

Consolidated Framework for Implementation Research/Reach, Effectiveness, Adoption, Implementation, Maintenance.

Using Consolidated Framework for Implementation Research, intervention characteristics (credible, low-cost MgSO_4_ bundle), inner setting (Emergency Department/L&D integration, staffing), and process (co-design, Plan–Do–Study–Act cycles, debriefs) shape execution; facilitation and leadership visibly normalize the ≤60-min goal (Birth, 2022; Institute for Healthcare Improvement. AIM Patient Safety Bundles, 2025). In Reach, Effectiveness, Adoption, Implementation, Maintenance terms—Reach (proportion of indicated patients receiving MgSO_4_/remote BP), Effectiveness (timeliness, SMM, eclampsia, readmissions), Adoption (order-set/BPA use across units), Implementation (fidelity to checklists/escalation), and Maintenance (governance and quarterly refreshers)—provide a durable evaluation frame (Countouris et al., 2025; Institute for Healthcare Improvement. AIM Patient Safety Bundles, 2025; World Health Organization).

Playbook (close)

1. Leadership sets the bar (≤60 min) and designates accountable owners for L&D, ED, and postpartum units.
2. Build one order set and one BPA, align MEOWS triggers, and pre-stage MgSO_4_ kits.
3. Run simulations and use bedside checklists to hard-wire monitoring and handoffs.
4. Stand up dashboards with equity stratification and a monthly learning loop to review delays, toxicity, and countermeasures—then iterate.

## 8 Competing schools of thought and ongoing controversies

Building on the above, several persistent areas of debate continue to shape real-world MgSO_4_ practice. Key questions include how tightly to target a numeric therapeutic range, how to balance clinical *versus* laboratory monitoring, how to individualize dosing in renal impairment and extreme BMI, and how far to extend MgSO_4_ beyond obstetrics into perioperative and ICU care.

### 8.1 Monitoring philosophy: “clinical reflex-first” vs. “lab-first”

A long-standing philosophical divide in MgSO_4_ practice concerns whether bedside clinical examination alone is sufficient for safety monitoring or whether routine serum magnesium levels should be mandated. Many obstetric programs, and several contemporary guidelines, explicitly favor a “clinical reflex–first” approach: deep-tendon reflexes, respiratory rate, and urine output are assessed at the bedside according to standardized observation charts, while serum magnesium levels are obtained only when renal function is impaired, infusions are prolonged, body habitus is extreme, or the examination is equivocal (National Institute for Health and Care Excellence NICE, 2019; Society of Obstetric Medicine of Austr alia and New Zealand SOMANZ, 2023). Recent Australasian guidance recommends scheduled bedside observations and discourages routine levels unless renal dysfunction is present or toxicity is suspected, and the United Kingdom National Institute for Health and Care Excellence guideline NG133 (*Hypertension in pregnancy*) guideline similarly centers monitoring on clinical signs within hypertensive-disorder pathways (National Institute for Health and Care Excellence NICE, 2019; Society of Obstetric Medicine of Austr alia and New Zealand SOMANZ, 2023). International consensus documents further urge each unit to maintain a uniform protocol for MgSO_4_ use and monitoring, with clinical observation and outcome auditing as the cornerstone (Magee et al., 2022a). This stance is coherent with the exposure–toxicity sequence summarized in Section 4.4 and the “clinical-first, level-supported” strategy outlined in Sections 4.5 and 6.4.

In contrast, some institutions adopt a more “lab–first” philosophy, advocating scheduled serum magnesium measurements to “hit” a predefined therapeutic window and reduce interindividual variability in exposure. Proponents point to heterogeneous pharmacokinetics in pregnancy, obesity, diuretic use, and renal impairment, arguing that fixed-rate infusions can produce subtherapeutic or toxic concentrations in a minority of patients (National Institute for Health and Care Excellence NICE, 2019; Society of Obstetric Medicine of Austr alia and New Zealand SOMANZ, 2023). From this perspective, routine levels are viewed as a way to standardize practice, address medico-legal concerns, and compensate for variable skill in reflex assessment. However, direct evidence that universal level monitoring improves maternal or neonatal outcomes over high-quality clinical surveillance is limited, and lab-based strategies may be constrained by turnaround times, resource use, and feasibility in low-resource settings.

Syntheses of current guidelines and consensus statements therefore lean toward a pragmatic compromise: clinical signs remain the primary real-time safety anchor for all patients, while serum magnesium levels are used selectively in higher-risk situations—impaired or deteriorating renal function, nonstandard or prolonged regimens, extremes of BMI, equivocal examinations, or co-therapies that plausibly alter magnesium handling (Magee et al., 2022a; National Institute for Health and Care Excellence NICE, 2019; Society of Obstetric Medicine of Austr alia and New Zealand SOMANZ, 2023). Within this hybrid model, units are encouraged to adopt a single, explicit monitoring protocol and to audit both processes (e.g., completeness of RR/DTR/UO documentation) and outcomes, rather than choose between reflex-only and lab-only extremes. The practical implementation of this compromise is detailed in Section 6.4, and consolidated action steps are summarized in Box 1 (Practice checklist).

### 8.2 Choice of regimen and dose–duration: Pritchard, Zuspan, and local variants

A recurring question is whether one MgSO_4_ regimen family should be regarded as the global “standard,” or whether multiple context-adapted regimens can be considered equivalent. Historically, the IM Pritchard regimen (IV loading followed by IM maintenance) has been favored where infusion pumps are scarce, whereas the IV Zuspan regimen (IV loading plus continuous 1–2 g/h infusion) is preferred in settings with pumps and continuous monitoring (National Institute for Health and Care Excellence NICE, 2019; Diaz et al., 2023). The 2023 Cochrane update comparing alternative regimens found broadly similar seizure outcomes across standard families (Pritchard, Zuspan, reduced-dose variants), with between-regimen differences driven more by feasibility and adverse-effect profiles than by clear efficacy separation; IM strategies carry injection-site pain and occasional abscess, whereas continuous IV regimens require reliable pumps and nursing vigilance (Diaz et al., 2023). These data support the view that multiple regimens can be acceptable, provided that recommended loading doses are delivered and basic monitoring is in place.

A related debate concerns how far dose and duration can be simplified. Low-dose variants such as the Dhaka regimen aim to reduce toxicity and resource demands, and several observational and trial data suggest that, in selected populations, lower maintenance doses and shorter postpartum courses (e.g., 12 vs. 24 h) do not clearly increase eclampsia risk (Diaz et al., 2023; Shaheen et al., 2024; Quist-Nelson et al., 2024; Begum et al., 2001). Critics note that most studies are underpowered for rare outcomes and conducted in specific settings, raising questions about generalizability; some guideline panels therefore remain cautious about endorsing aggressive dose reductions or very short durations as default care (Magee et al., 2022a; National Institute for Health and Care Excellence NICE, 2019). The balance between avoiding underexposure (breakthrough seizures) and minimizing cumulative dose and monitoring burden is particularly delicate in women with renal impairment or extreme BMI (Section 6.5).

From an implementation perspective, a pragmatic compromise is emerging. Rather than seeking a single universally superior regimen, many experts advocate that each institution or network (i) adopt one primary regimen family (Pritchard- or Zuspan-based) plus, where needed, a clearly defined alternative; (ii) ensure that loading doses are standardized and maintenance options (IV vs. IM, 12 vs. 24 h) are explicitly linked to local resources and patient risk; and (iii) monitor for breakthrough seizures, toxicity, and feasibility over time (Magee et al., 2022a; Diaz et al., 2023). Section 5.1.3 summarizes practical route and regimen choices in resource-constrained settings, while Box 1 distills key action points for regimen selection and program support.

### 8.3 Global diversity in neuroprotective dosing and timing

Published evidence explicitly characterizing global diversity in antenatal MgSO_4_ neuroprotection dosing and timing is limited; available professional guidance therefore suggests relatively low diversity in core practice, with most protocols converging on fixed loading-plus-maintenance regimens when preterm birth is imminent before approximately 30–32 weeks’ GA, while regional variation occurs mainly at the margins (e.g., gestational-age cutoffs and repeat-dose policies) (Crowther et al., 2023; Hywel Dda University Health Board, 2024). UK/Wales guidance operationalizes a practical window—ideally starting ∼4 h before birth but recommending administration even when the interval is shorter—reflecting real-world labor dynamics (Hywel Dda University Health Board, 2024). Notably, the MAGENTA randomized trial at 30–34 weeks evaluated a loading-dose–only (bolus-only) regimen (without a maintenance infusion) and reported no significant reduction in death or CP at 2 years; this hypothesis-generating finding raises the possibility that shorter bolus-only exposure may be insufficient for neuroprotection at later gestations, tempering enthusiasm for routine use beyond ∼30–32 weeks and reinforcing jurisdictional variability above that threshold (Crowther et al., 2023). Implementation programs such as Prevention of Cerebral Palsy in PreTerm Labour show that standardized prompts, checklists, and training can increase appropriate MgSO_4_ use without major safety signals, although effects depend on local baseline performance and fidelity (Edwards et al., 2025).

### 8.4 Sequencing MgSO_4_ with rapid antihypertensive therapy

In acute severe hypertension (≥160/110 mmHg) or high eclampsia risk, two time-critical tasks compete for attention: seizure prophylaxis or treatment with MgSO_4_ and rapid BP control with fast-acting antihypertensives (e.g., IV labetalol, IV hydralazine, oral immediate-release nifedipine; IV nitroglycerin when pulmonary edema is present) (National Institute for Health and Care Excellence NICE, 2019; Countouris et al., 2025). Traditional teaching in some settings emphasized “magnesium first” to prioritize seizure prevention, whereas other teams focused on “BP first” to reduce intracerebral hemorrhage risk. Systems-oriented statements and recent expert reviews now converge on a two-track strategy, in which MgSO_4_ and antihypertensives are initiated in close succession or in parallel whenever feasible, rather than sequentially in a way that delays either component (Edwards et al., 2025; National Institute for Health and Care Excellence NICE, 2019; Countouris et al., 2025).

The controversy is driven less by biology than by workflow and safety concerns. Advocates of a magnesium-first sequence note that eclampsia can occur before antihypertensive therapy takes effect and that MgSO_4_ has limited impact on systemic BP at obstetric doses (Countouris et al., 2025; Magley et al., 2025). Others worry that starting MgSO_4_ in a patient with uncontrolled severe hypertension and limited monitoring could blunt neurologic assessment or contribute to hypotension if antihypertensives are then rapidly escalated (Countouris et al., 2025). Direct comparative data on different sequencing strategies are sparse, and most reports bundle both interventions within broader emergency-response pathways, making it difficult to isolate the independent effect of order.

A pragmatic synthesis is to treat sequencing as a coordination problem rather than a rigid rule. The shared principle is that both therapies should be delivered rapidly, by clearly assigned team members, with continuous bedside monitoring of mental status, respiratory rate, DTRs, and BP (National Institute for Health and Care Excellence NICE, 2019). In practice, whichever intervention can be started safely first—for example, an oral nifedipine dose while IV access is established, or an MgSO_4_ loading dose while antihypertensive orders are being prepared—should not be delayed, provided that the second arm follows promptly. Operational details, including time-bound BP targets and bundle design, are described in Sections 5.4 and 7.2–7.3; Section 11 reframes these combined actions as core maternal-safety priorities.

### 8.5 MgSO_4_ as an adjunct sedative/analgesic in the OR/PACU/ICU

Beyond obstetrics, perioperative and ICU data suggest that IV MgSO_4_ can reduce peri- and postoperative analgesic needs, opioid consumption, and postoperative nausea and vomiting, with occasional reports of delayed emergence—effects attributed to NMDA antagonism and calcium-channel actions (Yue et al., 2022). Randomized trials also report less emergence agitation and reduced intraoperative remifentanil use when MgSO_4_ is co-administered, though effect sizes vary by procedure and background multimodal analgesia (Su et al., 2023). However, neutral observational findings exist (e.g., urologic cohorts without clear PACU pain benefit), underscoring context dependence (Salevitz et al., 2024). Clinically salient drug interactions include potentiation and prolongation of nondepolarizing neuromuscular blockers (notably rocuronium), mandating quantitative neuromuscular monitoring and appropriate reversal per American Society of Anesthesiologists 2023 guidance; additive sedation, bradycardia, and hypotension may emerge with propofol, dexmedetomidine, benzodiazepines, and opioids, requiring dose titration and vigilance (Thilen et al., 2023).

### 8.6 Synthesis: where we agree, where we disagree, what to do now

There is broad agreement that (i) MgSO_4_ is first-line for eclampsia prophylaxis and treatment and for fetal neuroprotection when very preterm birth is imminent; (ii) bedside clinical monitoring is foundational; and (iii) regimen choice should fit resources while achieving therapeutic exposure without toxicity. Debate persists over universal lab monitoring vs. selective levels, specific regimen superiority (IM vs. IV vs. hybrid), upper GA boundaries and repeat neuroprotection courses, and the net perioperative/ICU benefit across procedures and co-sedatives.

Practically, programs should (a) embed a reflex-first monitoring protocol with triggers for serum levels (renal impairment, prolonged infusion, atypical exams); (b) select IV or IM maintenance to match equipment and staffing; (c) align neuroprotection with local GA policy and “give even if < 4 h” when delivery is imminent; (d) ensure antihypertensive treatment within 30–60 min alongside MgSO_4_; and (e) in the OR/ICU, apply quantitative neuromuscular monitoring and hemodynamic vigilance when co-administering MgSO_4_ with sedatives and analgesics.

## 9 Current research gaps

### 9.1 PK–PD gaps in special populations

Despite routine use, contemporary PK–PD data for MgSO_4_ remain sparse in clinically complex obstetric populations—those with renal dysfunction, obesity/extreme BMI, multiple gestation, and ICU-level comorbidities (e.g., sepsis, cardiopulmonary disease). Recent population PK work in preeclampsia confirms substantial interindividual variability and identifies weight and disease severity as covariates, but many models lack CrCl or estimated glomerular filtration rate (eGFR), albumin, and explicit maternal–fetal compartments needed to inform exposure targets across settings (Deng et al., 2024). Future models should prospectively incorporate dynamic renal function, fluid shifts, and fetal transfer to link concentrations with both seizure suppression and toxicity end points. Standardized exposure–response outcomes—loss of deep-tendon reflexes, respiratory-rate thresholds, seizure recurrence—should be embedded alongside serum magnesium (total and, where feasible, ionized) to enable model-informed dosing. The latest preeclampsia PK study demonstrates the feasibility of such designs but underscores remaining covariate gaps and the need for simulation-tested regimens beyond “one-size-fits-all” programs (Deng et al., 2024). Renal-handling reviews further emphasize the predominance of renal clearance and the sharp rise in toxicity risk with reduced GFR, yet pregnancy-validated dose-adjustment algorithms are lacking (Adomako and Yu, 2024).

### 9.2 Head-to-head regimen trials

Modern, adequately powered randomized trials directly comparing Pritchard (IV load + IM maintenance), Zuspan (IV load + continuous infusion), and hybrid or bolus-dominant dosing—using standardized workflow and toxicity outcomes—are scarce. The updated Cochrane comparison of alternative regimens found no high-certainty evidence that any single approach is superior for major maternal or perinatal outcomes, reflecting small, heterogeneous studies and underscoring the need for pragmatic, multicountry head-to-head trials stratified by resource level and infusion-pump availability (Diaz et al., 2023).

### 9.3 Drug–drug interaction quantification in perioperative/ICU care

Perioperative and ICU data suggest anesthetic- and analgesic-sparing effects with MgSO_4_, but the magnitude of interactions with propofol, dexmedetomidine, midazolam, and opioids remains incompletely quantified for obstetric populations (onset, duration, dose–response, and hemodynamic trade-offs). High-quality randomized evidence confirms potentiation and prolongation of nondepolarizing neuromuscular block (e.g., rocuronium), yet recovery kinetics and reversal dosing are still insufficiently characterized for cesarean and postpartum ICU cohorts, arguing for obstetric-specific trials with quantitative neuromuscular monitoring and prespecified safety end points (bradycardia, hypotension, delayed emergence) (Benette et al., 2025).

### 9.4 Long-term outcomes after antenatal exposure

The neuroprotective effect of antenatal MgSO_4_ on CP is consistent, but longer-term neurodevelopment beyond 2 years is underreported, and benefits at later GA remain uncertain. The 2024 synthesis highlights reductions in CP and death/CP but calls for longer-term follow-up and better generalizability to LMIC settings (Shepherd et al., 2024). At 30–34 weeks’ gestation, the MAGENTA randomized trial showed no significant effect on death or CP at 2 years, underscoring the need to refine gestational-age windows and to power studies for clinically meaningful differences in contemporary care (Crowther et al., 2023). Notably, MAGENTA evaluated a loading-dose–only (bolus-only) regimen without a maintenance infusion; future studies should therefore explicitly test regimen design and exposure duration (e.g., bolus-only vs. longer-exposure strategies) to determine whether insufficient exposure contributes to the lack of observed benefit at later gestations (Crowther et al., 2023). Lactation safety summaries indicate very low infant exposure and generally reassuring clinical experience, but systematic pharmacovigilance beyond the early neonatal period is limited, and harmonized reporting of breastfeeding outcomes is needed [Drugs and Lactation Database (LactMed®), 2006)].

### 9.5 Prediction algorithms and early-warning systems

Digitized maternity early-warning scores (MEWS/MEOWS) are being developed nationally, yet external validation, calibration drift, and transportability across hospitals and subgroups (e.g., hypertensive disorders) remain major gaps. UK national score development work explicitly identified the need for prospective validation and impact evaluation before widespread adoption, including delineation of escalation pathways that integrate timely MgSO_4_ initiation (Gerry et al., 2024). Future studies should report subgroup performance (preeclampsia/eclampsia, obesity, renal impairment), fairness metrics, and workflow impacts (false-alarm burden vs. time-to-treatment gains).

### 9.6 Implementation and economics

Health-system bundles (order sets, nursing checklists, EHR alerts) are widely promoted to accelerate treatment of severe hypertension and ensure timely MgSO_4_ administration, but formal cost-effectiveness and budget-impact evaluations of these MgSO_4_-inclusive bundles are rare. Available economic frameworks from broader hypertensive-disorder packages (e.g., Community-Level Interventions for Pre-eclampsia program analyses in LMICs) offer templates—choice of perspective, time horizon, and inclusion of maternal strokes prevented, SMM, neonatal ICU days, and readmissions—that should be adapted to facility-level MgSO_4_ implementation, with equity-stratified reporting (Van Iseghem et al., 2025).

### 9.7 Cross-cutting data standards needed

Across domains, consensus core outcome sets should be adopted: (1) exposure metrics (total and ionized magnesium, timing relative to delivery); (2) maternal clinical outcomes (eclampsia, recurrent seizures, reflex/respiratory toxicity, need for calcium rescue, ICU transfer); (3) neonatal outcomes (CP, severe intraventricular hemorrhage, ventilation days, NICU length of stay, breastfeeding continuity); and (4) implementation outcomes (time-to-treatment, adherence, alarm/alert acceptance). Studies should preregister analysis plans, publish model code and dosing simulators, and commit to multisite external validation.

### 9.8 Research agenda (priority actions)

#### 9.8.1 Adaptive, multi-country regimen trials

Pragmatic RCTs in high- and low-/middle-income settings comparing IV, IM, and hybrid dosing, powered for seizures, toxicity, and workflow outcomes, with prespecified resource-fit algorithms and quantitative monitoring (Diaz et al., 2023).

#### 9.8.2 Population PK–PD consortia

Prospective modeling across renal dysfunction, obesity, multiple gestation, and ICU comorbidity, capturing CrCl/eGFR, albumin, fluid balance, and maternal–fetal transfer; publication of simulators to guide individualized dosing and therapeutic drug-monitoring triggers (Deng et al., 2024; Adomako and Yu, 2024).

#### 9.8.3 Interaction quantification

Obstetric-specific RCTs measuring MgSO_4_ interactions with rocuronium and sedatives/analgesics, including onset/offset curves, reversal dosing, hemodynamics, and residual block in cesarean and postpartum ICU cohorts (Benette et al., 2025).

#### 9.8.4 Long-term and lactation outcomes

Harmonized follow-up beyond 2 years for neuroprotection cohorts and prospective breastfeeding pharmacovigilance registries incorporating infant neurodevelopment and readmissions [Shepherd et al., 2024; Crowther et al., 2023; Drugs and Lactation Database (LactMed®), 2006)].

#### 9.8.5 Implementation economics

Trial-embedded cost-effectiveness and budget-impact analyses of MgSO_4_ bundles (order sets, alerts, checklists) with equity-stratified outcomes, leveraging established maternal-safety frameworks (Van Iseghem et al., 2025).

## 10 Future directions

### 10.1 Precision dosing *via* model-informed and Bayesian adaptation

An immediate opportunity is a model-informed precision dosing (MIPD) pathway that individualizes MgSO_4_ to renal function, body size, and dynamic fluid shifts. Recent popPK work in preeclampsia demonstrates substantial interindividual variability and supports simulation-based dose optimization; future models should routinely include eGFR/CrCl, BMI, serum albumin, and time-varying fluid balance, with explicit maternal–fetal compartments to link exposure with seizure suppression and toxicity end points (Deng et al., 2024). Given the predominance of renal elimination and the rapid escalation of toxicity with reduced GFR, integrating kidney function into dosing logic is essential (Adomako and Yu, 2024). At the bedside, sparse sampling (for example, 1–2 serum magnesium levels during maintenance) can feed Bayesian updates to refine infusion rates in real time—an approach widely described for therapeutic drug monitoring–to–MIPD transitions in other domains and readily portable to MgSO_4_ (Wicha et al., 2021). Clinically, this pathway should be embedded in the EHR: a standardized order set (loading and maintenance), a covariate capture panel (weight, eGFR, urine output), exposure targets (for example, a therapeutic band defined by seizure-prophylaxis vs. toxicity thresholds), and a decision-support widget that ingests a single level and proposes a revised rate with guardrails for renal impairment (Deng et al., 2024; Adomako and Yu, 2024; Wicha et al., 2021).

### 10.2 Pragmatic multi-center trials

Conventional trials remain necessary but should adopt pragmatic designs that reflect real-world workflows. Stepped-wedge or cluster-randomized rollouts across L&D, ED, and postpartum units can test “dose-to-target” algorithms or regimen families (IV vs. IM vs. hybrid) using co-primary outcomes: (i) time-to-target BP (initiation within 30–60 min) and (ii) composite maternal–fetal outcomes (eclampsia or ICU transfer; neonatal severe morbidity), with equity stratification by race/ethnicity, language, and rurality (Countouris et al., 2025). Registry-embedded trials can leverage existing severe-hypertension pathways and early-warning systems to capture fidelity, escalation timing, and adverse events at scale (Countouris et al., 2025; Gerry et al., 2024). To complement neuroprotection evidence, new trials should oversample LMIC sites and specify longer-term child outcomes beyond 2 years, responding to calls from recent systematic reviews for durable neurodevelopmental follow-up and broader generalizability (Shepherd et al., 2024).

### 10.3 Physiologic biomarkers and AI toxicity alerts

Digitized maternity early-warning scores (MEWS/MEOWS) provide a validated foundation for algorithmic surveillance and escalation (Gerry et al., 2024). Next-generation systems should fuse continuous vital-sign streams (respiratory rate, oxygen saturation), urine output, and neuromuscular monitoring (when nondepolarizing blockers are co-administered) to detect impending MgSO_4_ toxicity—e.g., rising apnea risk or loss of reflexes—while simultaneously prompting antihypertensive action when severe BP is sustained (Countouris et al., 2025; Gerry et al., 2024). After discharge, remote postpartum BP programs can extend this safety net, enabling algorithm-driven triage and rapid treatment of late-worsening hypertension; such programs have demonstrated feasibility and improved capture of at-risk patients in safety-net populations (Mujic et al., 2024). Any AI or alert feature must undergo prospective validation, include drift monitoring, and maintain human-in-the-loop oversight to avoid automation bias and alert fatigue (Countouris et al., 2025; Gerry et al., 2024; Mujic et al., 2024).

### 10.4 Interdisciplinary collaboration and governance

Future practice should codify shared governance across obstetrics, anesthesia, ICU, and neonatology for MgSO_4_ co-administration. Standard operating procedures need to define handoffs (admission → delivery → PACU/ICU → postpartum), neuromuscular monitoring rules when MgSO_4_ is combined with rocuronium or other nondepolarizing agents, and reversal workflows consistent with 2023 American Society of Anesthesiologists guidance (quantitative monitoring and timely antagonism) (Thilen et al., 2023). Service-level agreements should specify escalation timelines (e.g., treatment within 30–60 min), documentation requirements, and a cross-service debrief process after any seizure, toxicity rescue, or ICU transfer (Thilen et al., 2023; Countouris et al., 2025).

### 10.5 Global health adaptation and QI-driven scalability

Scale-up will depend on a “minimum viable bundle” that functions with or without infusion pumps: a single standardized order set (with IV and IM branches), a bedside checklist (reflexes, respirations, urine output), an escalation pathway for severe BP, and a simple data form for timeliness and outcomes. This starter bundle can begin on paper and transition to the EHR as capacity grows, following the national-to-facility architecture promoted by the WHO QCN (coaching, micro-audits, and iterative learning) (World Health Organization). Cost-effectiveness should be embedded in implementation studies; extended evaluations of national programs integrating antenatal MgSO_4_ have shown that effectiveness and cost-effectiveness can be appraised together, informing procurement and workforce planning for sustained adoption (Edwards et al., 2025). Where remote monitoring is feasible, postpartum BP programs should be bundled with MgSO_4_ pathways and audited for equity—ensuring that gains are shared across language and rurality strata (Countouris et al., 2025; Mujic et al., 2024).

### 10.6 One-slide roadmap (in prose)

Data → Model → Bedside tool → Trial → Scale-up: collect covariates (eGFR, BMI, fluid balance) and sparse levels → develop popPK models with Bayesian updating and exposure targets → build EHR decision support that proposes maintenance rate adjustments → conduct cluster or registry trials measuring timeliness and maternal–fetal outcomes with equity analyses → scale nationally *via* QI collaboratives and WHO QCN, with embedded cost-effectiveness (Edwards et al., 2025; Deng et al., 2024; Adomako and Yu, 2024; Countouris et al., 2025; Gerry et al., 2024; World Health Organization; Wicha et al., 2021).

### 10.7 Design sketch (pragmatic trial)

Adults with preeclampsia/eclampsia in hospital clusters (mixed acuity) would be randomized by stepped-wedge design to MIPD-guided *versus* standard MgSO_4_. Primary endpoints: initiation within 30–60 min and an eclampsia/ICU-transfer composite by 48 h. Secondary endpoints: toxicity rescue events, neonatal severe morbidity, and length of stay, with 6–24-month neurodevelopmental follow-up in a predefined subgroup. Equity analyses would use interaction terms for race/ethnicity, language, and rurality (Shepherd et al., 2024; Countouris et al., 2025).

### 10.8 MIPD quick list

#### 10.8.1 Key covariates

eGFR/CrCl, BMI, albumin, urine output, fluid balance, gestational age. Suggested sampling times: post-load (30–60 min) and during maintenance (2–4 h) where feasible. Target band: a pragmatic therapeutic range anchored to seizure-prevention and toxicity thresholds, configurable by site. Bayesian cadence: one update per patient episode unless renal function or clinical status changes (Deng et al., 2024; Adomako and Yu, 2024; Wicha et al., 2021).

#### 10.8.2 AI/alert safety note

AI- and alert-based tools should be validated at external sites, with ongoing monitoring of calibration and false-alarm burden. Every alert should have named clinical ownership, clear documentation of actions taken, and versioned provenance logs. Retraining should incorporate explicit drift checks at predefined intervals (Countouris et al., 2025; Gerry et al., 2024; Mujic et al., 2024).

#### 10.8.3 Global scale-up note

Global dissemination should align with WHO QCN coaching and audit tools, using open-access order-set and checklist templates. Budget-impact and cost-effectiveness analyses should explicitly include maternal strokes prevented, ICU days, and postpartum readmissions to inform national-scale decisions (Edwards et al., 2025; World Health Organization).

## 11 Practice and policy implications

### 11.1 Clinical practice priorities

Clinical and institutional leaders should approach MgSO_4_ as part of an integrated maternal-safety bundle rather than an isolated drug protocol. Priority actions are to: recognize acute severe hypertension rapidly; initiate treatment within 30–60 min; and start MgSO_4_ in parallel when indicated. Bedside monitoring should consistently use the triad of respiratory rate, deep-tendon reflexes, and urine output, with explicit thresholds for holding or adjusting therapy. Order sets and handoff checklists should incorporate context-aware dosing and duration that reflect renal function, body size, gestational age, and care setting. For threatened early preterm birth, antenatal MgSO_4_ should be operationalized as a routine neuroprotection step once birth is likely, without delaying indicated delivery. Box 1 summarizes practice-ready checklists for acute hypertension, seizure prophylaxis, fetal neuroprotection, and perioperative/ICU guardrails.

BOX 1 **Practice checklist.**

Key MgSO_4_ priorities across obstetric and critical-care settings.

1. Acute severe hypertension (with or without preeclampsia)
  - Recognize and time-stamp acute severe hypertension (e.g., systolic blood pressure ≥160 mmHg or diastolic blood pressure ≥110 mmHg sustained ≥15 min) as an emergency.
  - Treat within 30–60 min using recommended antihypertensives and start MgSO_4_ in parallel when eclampsia or preeclampsia with severe features is present.
  - Ensure bedside monitoring of respiratory rate, deep-tendon reflexes, and urine output (RR/DTR/UO) with clear thresholds for holding, adjusting, and escalating.
2. Eclampsia/preeclampsia with severe features—anticonvulsant regimen
  - Use MgSO_4_ as first-line anticonvulsant for seizure control and prophylaxis.
  - Choose a protocol (e.g., Zuspan IV infusion or Pritchard IM regimen) that matches local staffing, monitoring capacity, and drug-preparation workflow.
  - Continue therapy for an appropriate duration after the last seizure or delivery, with planned reassessment rather than arbitrary stopping.
3. Antenatal MgSO_4_ for fetal neuroprotection
  - Offer MgSO_4_ neuroprotection routinely when very preterm birth is imminent (strongest evidence <30 weeks; individualized 30–34 weeks per policy).
  - Avoid delaying indicated delivery to complete an arbitrary infusion time; prioritize timely birth and team coordination.
  - Embed neuroprotection into standardized preterm-birth pathways with neonatal team notification and structured documentation.
4. Perioperative/ICU adjunct use
  - Use MgSO_4_ as a co-analgesic or sedative adjunct only where hemodynamics, neuromuscular function, and recovery can be closely monitored.
  - Titrate cautiously, especially when combined with other sedatives or non-depolarizing neuromuscular blockers; follow quantitative NMB monitoring and reversal guidance.
  - In obstetric cases, pair MgSO_4_ with active postpartum hemorrhage precautions (Active Management of the Third Stage of Labor, uterotonics ready, quantitative blood loss measurement).
5. *Postartum* follow-up, safety, and equity
  - Guarantee postpartum blood-pressure reassessment and follow-up (in-person or remote) within locally defined time windows for women with HDP.
  - Track timeliness of treatment, MgSO_4_ completion, monitoring completeness, neuroprotection uptake, and postpartum follow-up as routine quality indicators.
  - Stratify these metrics by key equity dimensions (e.g., language, insurance, geography, ethnicity) and address gaps through targeted quality-improvement actions.

Expanded 90-day playbooks, operational cadence, and example KPI targets are provided in Supplementary Box S1 and Table 2.

### 11.2 Institutional systems, digital tools, and training

Durable reliability requires aligned tools, teams, and digital infrastructure. Standardized electronic order sets, well-designed alerts, and structured handoffs should be coupled with simulation-based team training, pharmacy support, and bedside nursing protocols so that MgSO_4_ bundles are delivered consistently across L&D, emergency, and ICU environments. Where electronic alerts are used, they should be validated for calibration and false-alarm burden, assigned clear clinical ownership, and monitored for drift over time, with explicit escalation rules and rollback plans. Example 90-day playbooks, operational cadence, and candidate KPI targets are provided in Supplementary Box S1 and can be adapted to local governance structures and resource constraints.

### 11.3 Measurement, equity, and scale-up

Measurement and equity need to be built in from the outset. Programs should track timeliness of treatment for acute severe hypertension, completion of MgSO_4_ regimens, monitoring completeness, antenatal neuroprotection uptake, and postpartum blood-pressure follow-up, using run charts and regular case review to identify gaps. Many quality-improvement collaboratives set pragmatic targets—for example, achieving timely treatment in at least 80%–90% of eligible cases within a few quarters—while using equity-stratified dashboards to ensure that progress is shared across language, insurance, and geographic subgroups. Table 2 presents a compact KPI panel that can be tailored to local priorities. At regional and national levels, aligning MgSO_4_ bundles with WHO QCN tools, publishing open templates for order sets and checklists, and incorporating budget-impact and cost-effectiveness analyses can support scale-up and sustained investment, particularly in resource-constrained settings.

**TABLE 2:** Metrics and equity (SDG-aligned KPI panel).

Category	KPI (definition and formula)	Targets (example)	Equity stratifiers and notes
A. Timeliness (process)	T1 – ≤60-min initiation: % acute severe BP episodes with time-zero → first antihypertensive ≤60 min. T2 – Parallel MgSO_4_ (when indicated): % eligible cases with antihypertensive and MgSO_4_ started within the same 60-min window.	T1/T2 – Timeliness: example target ≥80–90% of eligible episodes treated within 60 min.	Use the same equity stratifiers for all KPIs (e.g., language, insurance, ethnicity, site/region). Time zero is the first qualifying BP sustained ≥15 min. See Box 1 for indications.
B. Treatment completeness (process)	C1 – Regimen completion: % indicated cases receiving load + maintenance per protocol. C2 – First neuroprotection dose pre-birth: % eligible imminent preterm births receiving antenatal MgSO_4_ before delivery.	C1 – Completion: example target ≥90% of indicated cases. C2 – Neuroprotection: example target ≥80–90% of eligible imminent preterm births.	Use the same equity stratifiers. Ensure premix/stock availability and neonatal team pre-alert for antenatal MgSO_4_ courses.
C. Monitoring and safety (structure/process)	M1 – Bedside triad documented: % infusions with RR/DTRs/UO charted at the required cadence. M2 – Escalation timeliness: % meeting hold criteria with escalation within 15–30 min. M3 – Antidote availability: % areas where calcium gluconate is immediately available.	M1 – Monitoring completeness: example target ≥90% of infusions. M2 – Escalation timeliness: example target ≥90% of episodes. M3 – Antidote availability: example target 100% of relevant areas.	Use the same equity stratifiers. Hold/adjust thresholds include RR < 12/min, absent DTRs, UO < 25–30 mL/h (see Box 1 for details).
D. Continuity and outcomes (result)	R1 – *Postartum* BP follow-up: % HDP cases with in-hospital BP check ≤72 h and contact within 7–10 days after discharge (remote follow-up acceptable per policy). R2 – Safety outcomes: 30-day readmission/ED revisit; documented PPH/transfusion safeguards in obstetric cases.	R1 – *Postartum* follow-up: example target ≥80–90% of HDP cases receiving follow-up within the defined window. R2 – Safety outcomes: aim to maintain rates ≤ baseline; sustained spikes should trigger special-cause review.	Use the same equity stratifiers. Document AMTSL, readiness of uterotonics, and quantitative blood loss as safeguards against PPH.

Time zero is the first qualifying BP (SBP ≥160 or DBP ≥110) sustained ≥15 min. KPI, targets shown are illustrative, based on typical quality-improvement collaboratives, and should be adapted to local baseline performance, resources, and governance structures. Equity stratifiers are also illustrative and should be tailored to locally relevant dimensions (e.g., language, insurance type, geography, ethnicity).

Abbreviations: AMTSL, active management of the third stage of labor; BP, blood pressure; DTRs, deep tendon reflexes; ED, emergency department; HDP, hypertensive disorders of pregnancy; PPH, postpartum hemorrhage; RR, respiratory rate; UO, urine output.

## 12 Conclusion

MgSO_4_ has evolved from an anticonvulsant for eclampsia into a multidimensional therapy spanning seizure prophylaxis, fetal neuroprotection, and perioperative or ICU adjunct use. Across these indications, a coherent picture emerges: MgSO_4_ is highly effective when appropriately dosed, yet exposure varies widely with renal function, body size, and intercurrent illness. Clinical monitoring of respiratory rate, deep-tendon reflexes, and urine output remains the safest anchor for toxicity surveillance, with serum levels reserved for uncertainty, extremes of dosing, or organ dysfunction. When embedded in standardized bundles for acute severe hypertension, threatened preterm birth, and high-risk obstetric anesthesia, MgSO_4_ can substantially reduce preventable maternal and neonatal harm.

Future progress depends on integrating pharmacology, implementation science, and equity. Priority agendas include generating robust PK–PD and safety data in under-studied groups (e.g., obesity, kidney disease, critical illness); refining indication-specific dosing and duration with model-informed and trial-based approaches; and clarifying the role of MgSO_4_ as an adjunct in perioperative and ICU care beyond obstetrics. In parallel, health systems should hard-wire MgSO_4_ into maternal-safety bundles, track timeliness and monitoring with equity-stratified KPIs, and align local protocols with international quality-of-care initiatives. Closing these gaps would move MgSO_4_ practice from historical habit toward precise, accountable, and globally equitable use.

## Glossary

**AIM**: Alliance for Innovation on Maternal Health
**BMI**: body mass index
**BP**: blood pressure
**BPA(s)**: Best Practice Advisory/Advisories
**CP**: cerebral palsy
**CrCl**: creatinine clearance
**DTR(s)**: deep-tendon reflex(es)
**eGFR**: estimated glomerular filtration rate
**EHR**: electronic health record
**GA**: gestational age
**GFR**: glomerular filtration rate
**HDP**: hypertensive disorders of pregnancy
**HELLP**: hemolysis, elevated liver enzymes, low platelets
**ICU(s)**: intensive care unit(s)
**IM**: intramuscular
IL-1β: interleukin-1 beta
**IV**: intravenous
**KPI(s)**: key performance indicator(s)
**L&D**: Labor and Delivery
**LMIC(s)**: low- and middle-income country/countries
**MEOWS**: Modified Early Obstetric Warning System
**MEWS**: Modified Early Warning Score
**Mg** ^**2+**^: magnesium ion
**iMg** ^**2+**^: ionized magnesium
**MgSO**_**4**_: magnesium sulfate
**MIPD**: model-informed precision dosing
**NICU**: neonatal intensive care unit
**NMB(s)**: nondepolarizing neuromuscular blocker(s)
**NMDA**: N-methyl-D-aspartate
NF-κB: nuclear factor kappa B
**OR(s)**: operating room(s)
**PACU(s)**: post-anesthesia care unit(s)
**PK**: pharmacokinetics/pharmacokinetic
**PK–PD**: pharmacokinetic–pharmacodynamic
**popPK**: population pharmacokinetics
**QCN**: Quality of Care Network
**QI**: quality improvement
**RCT(s)**: randomized controlled trial(s)
**RR**: respiratory rate
**SMM**: severe maternal morbidity
**UO**: urine output
**WHO**: World Health Organization
