# Supplementary material for: Magnesium sulfate pharmacology for maternal and critical-care indications: mechanisms, pharmacokinetics, and the therapeutic window
Source: Front Pharmacol. 2026 Feb 20;17:1749828. doi: 10.3389/fphar.2026.1749828 (PMC12963761; doi:10.3389/fphar.2026.1749828)
Supplement: Supplementary file 1 [file Supplementaryfile1.docx]

Supplementary Box S1. Example 90-day playbook and operational cadence for magnesium sulfate bundles

| **A. 90-day implementation pathway**   - **Weeks 0–2: Baseline & aim setting**   - Audit recent cases of acute severe hypertension, eclampsia, antenatal neuroprotection, and perioperative MgSO₄ use.   - Quantify key gaps: time-to-treatment, missed or incomplete MgSO₄ regimens, missing RR/DTR/UO documentation, low neuroprotection uptake, weak postpartum follow-up.   - Agree on a focused aim statement (e.g., “Within 90 days, reliably treat ≥80% of acute severe hypertension episodes within 60 minutes and start indicated MgSO₄ in parallel”). - **Weeks 2–6: Tools, training, and pilot**   - Build or refine standardized order sets for acute severe hypertension, eclampsia prophylaxis/treatment, antenatal neuroprotection, and perioperative MgSO₄.   - Implement structured handoff templates that include time-stamped blood pressures, MgSO₄ doses, cumulative dose, and RR/DTR/UO status.   - Run brief multidisciplinary simulations (obstetrics, anesthesia, ICU, pharmacy, nursing) focusing on role clarity, escalation rules, and use of checklists.   - Pilot the updated bundles on one or two units (e.g., labor and delivery, obstetric HDU), with daily feedback to frontline teams. - **Weeks 6–12: Scale-up and refinement**   - Spread successful workflows to all relevant clinical areas (emergency, labor and delivery, postpartum wards, ICU).   - Track process indicators with unit-level run charts and rapid case reviews for outliers.   - Adjust alert thresholds, documentation prompts, and training frequency based on observed failure modes and staff feedback.   - Prepare a brief report to hospital leadership summarizing early impact, resource needs, and next-step commitments.   **B. Operational cadence for QI teams**   - **Monthly (or bi-monthly) review meetings**   - Review run charts for:     - timeliness of treatment for acute severe hypertension,     - MgSO₄ initiation when indicated,     - monitoring completeness (RR/DTR/UO),     - antenatal neuroprotection uptake among eligible births,     - postpartum blood-pressure follow-up.   - Stratify each metric by language, insurance type, geography, and other locally relevant equity dimensions.   - Select 2–3 outlier cases for rapid debrief and assignment of concrete action items (e.g., update order set, adjust alert logic, schedule refresher training). - **Team structure and roles**   - Designate a clinical lead (e.g., obstetrician or anesthesiologist), a nursing lead, and a data/IT representative as core owners of the MgSO₄ bundle.   - Clarify responsibilities for maintaining order sets, monitoring alert performance, preparing data for review, and reporting to leadership.   - Embed these tasks into existing governance structures (perinatal safety committee, ICU safety huddles, maternal-morbidity review meetings).   **C. Example KPI targets and equity guardrails**   - **Core process KPI examples**   - Timely treatment for acute severe hypertension (antihypertensive started within 60 min of qualifying blood pressure):     - pragmatic target: ≥80% of eligible episodes within the first quarter of implementation, ≥90% within two to three quarters.   - MgSO₄ initiation when indicated (eclampsia or preeclampsia with severe features): aim for ≥90% of eligible cases.   - Monitoring completeness (RR/DTR/UO) during MgSO₄ infusion: aim for ≥90% of intervals documented as per protocol.   - Antenatal MgSO₄ neuroprotection among eligible very preterm births: aim for ≥80–90%, adjusted for local case mix.   - Postpartum blood-pressure contact or visit within the locally defined window after discharge: aim for ≥80% initially, moving toward ≥90%. - **Equity and safety guardrails**   - Monitor every KPI in at least two equity cuts (e.g., by language and insurance status) and flag gaps >5–10 percentage points for targeted intervention.   - Set explicit thresholds for re-design (e.g., repeated failure to reach ≥70% in any subgroup over two to three review cycles triggers a structured root-cause analysis).   - Periodically review near misses, adverse events, and staff-reported concerns related to MgSO₄ (e.g., toxicity, delayed treatment, missed follow-up) to refine protocols.   *These figures are illustrative and should be adapted to local baseline performance, resources, and existing quality-improvement infrastructure.* |
| --- |
